# An enhanced version of Harris Hawks Optimization by dimension learning-based hunting for Breast Cancer Detection

**DOI:** 10.1038/s41598-021-01018-7

**Published:** 2021-11-09

**Authors:** Navneet Kaur, Lakhwinder Kaur, Sikander Singh Cheema

**Affiliations:** Department of Computer Science and Engineering, Punjabi University, Patiala, Patiala, 147002 India

**Keywords:** Breast cancer, Evolutionary theory

## Abstract

Swarm intelligence techniques have a vast range of real world applications.Some applications are in the domain of medical data mining where, main attention is on structure models for the classification and expectation of numerous diseases. These biomedical applications have grabbed the interest of numerous researchers because these are most serious and prevalent causes of death among the human whole world out of which breast cancer is the most serious issue. Mammography is the initial screening assessment of breast cancer. In this study, an enhanced version of Harris Hawks Optimization (HHO) approach has been developed for biomedical databases, known as DLHO. This approach has been introduced by integrating the merits of dimension learning-based hunting (DLH) search strategy with HHO. The main objective of this study is to alleviate the lack of crowd diversity, premature convergence of the HHO and the imbalance amid the exploration and exploitation. DLH search strategy utilizes a dissimilar method to paradigm a neighborhood for each search member in which the neighboring information can be shared amid search agents. This strategy helps in maintaining the diversity and the balance amid global and local search. To evaluate the DLHO lot of experiments have been taken such as (i) the performance of optimizers have analysed by using 29-CEC -2017 test suites, (ii) to demonstrate the effectiveness of the DLHO it has been tested on different biomedical databases out of which we have used two different databases for Breast i.e. MIAS and second database has been taken from the University of California at Irvine (UCI) Machine Learning Repository.Also to test the robustness of the proposed method its been tested on two other databases of such as Balloon and Heart taken from the UCI Machine Learning Repository. All the results are in the favour of the proposed technique.

## Introduction

**Figure 1:**
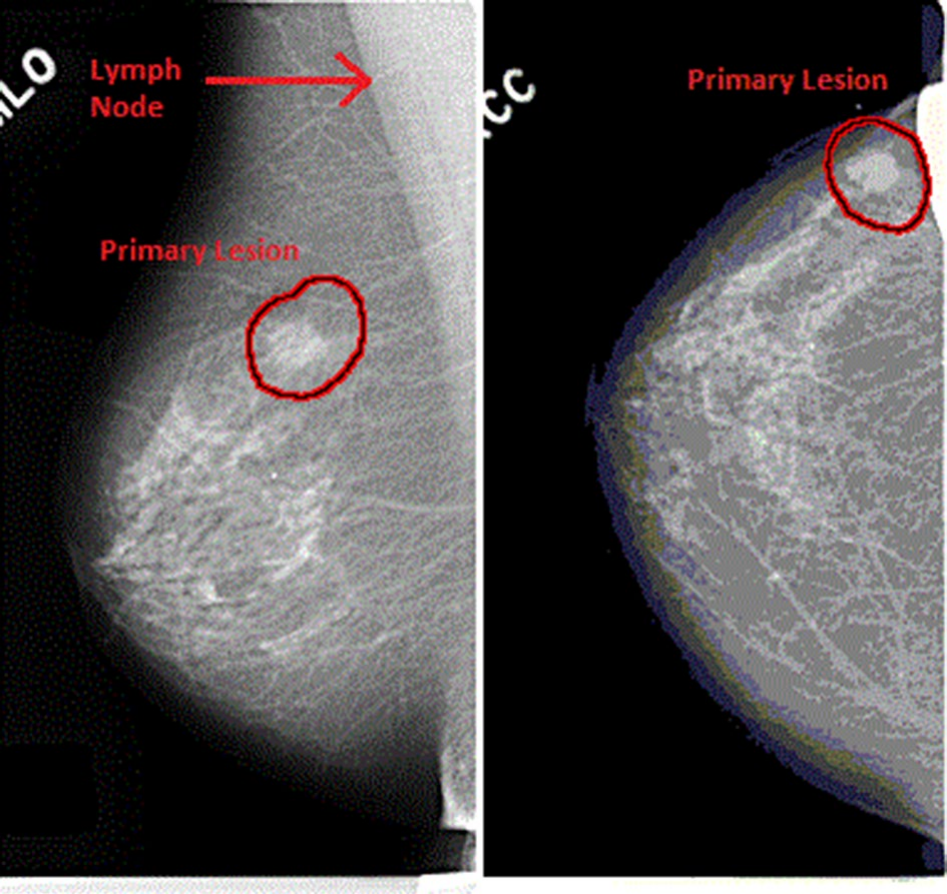
MLO and CC view of a mammogram.

Breast Cancer is the leading cause of deaths of women in all over the world and it happens to more than 8% of women in their lifetime^1^. The combination of medical science and technology contributes a lot to human health and their quality of life.It is common among women while rare among men. Breast cancer commonly affects women more than 40 years of age however younger women can also be affected especially with genetic predisposition (a genetic characteristic that influences the development of an individual organism under the influence of environmental conditions). It arises from the breast tissues mostly from the ductal carcinoma (the inner lining of milk ducts) or less frequently from the lobular carcinoma (the lobules that supply milk to the ducts). The risk factors for breast cancer are age, genetics, obesity, family history or late pregnancy^2^.Due to the factors related to cost and professional experience, in the last two decades computer systems to support detection and diagnosis have been developed in order to assist experts in early detection of abnormalities in their initial stages. Despite the large number of researches on computer-aided systems, there is still a need for improved computerized methods^3^.Breast cancer in men does not occur very often, less than 1% of all breast cancers occur in men. For men, the risk of being diagnosed with breast cancer during lifetime is about 1 in 1,000. Men and women all have breast tissues. The various hormones in women’s bodies exhilarate the breast tissue to grow into full breasts while men’s bodies normally don’t form much of the breast-stimulating hormones. As a result, their breast tissue usually stays flat and small.Mammography is the most widely used modality for detecting and characterizing breast cancer. It is a medical imaging that uses low-dose X-ray system to see inside the breasts. A mammography exam, called a mammogram, helps in the early detection and diagnosis of breast diseases. It has high sensitivity and specificity due to which small tumours and microcalcifications can be detected on mammograms. During mammography two views of each breast^4^ are recorded (see in Fig. 1):

- **Craniocaudal (CC) view** It is one of the two standard projections in a screening mammography. It is a top to bottom view that must show the medial part as well the external lateral portion of the breast as much as possible.
- **Mediolateral Oblique (MLO) view** It is a side view taken at an angle. The presence of pectoral muscle on the MLO view is a key component in acquiring the acceptability of the film. For reducing the false negatives resulting in increasing the sensitivity, the amount of visible pectoral muscle plays an important role.

Radiologists visually search mammograms for specific abnormalities. Some of the important signs of breast abnormalities that radiologists look for are:

- Calcifications that are tiny mineral deposits (calcium) scattered throughout the mammary gland, or occur in clusters. They appear as small bright spots on the mammogram. Concentration of microcalcifications in one place is also in favor of malignancy while the scattered calcifications are usually benign.
- Masses defined as a space occupying lesion. These are areas that look abnormal and they can be many things, including cysts (non-cancerous, fluid-filled sacs) and non-cancerous solid tumors (such as fibroadenomas) , but may sometimes may be a sign of cancer.
- Architectural distortions are when the normal architecture is distorted with no definite mass visible. This includes spiculations radiating from a point, and focal retraction or distortion of the edge of the parenchyma. It appears as a distortion in which surrounding breast tissues appear to be “pulled inward” into a focal point.

In the last few decades, many methods have been adopted for making such systems which can help in assisting radiologists in detecting any type of abnormalities in a mammogram for initial investigation. But due to large amount of data and their complexities no single algorithm is sufficient for that so we needed more enhanced versions for tackling these issues. Some works already done by the researchers have been described in this phase.

Tariq Sadad et al.^5^ proposed a CAD system in which segmentation technique i.e. FCMRG (Fuzzy C-mean Region Growing) is applied to obtain the mass in the image. Different features are extracted such as LBP-GLCM and LPQ (local Phase Quantization) and the feature selection is done on the basis of mRMR (minimum-redundancy maximum-relevancy) algorithm. At the end classification is carried out using different classifiers such as Decision Tree (DT), Support Vector Machine (SVM), Logistic Regression (LR), Linear Discriminant Analysis (LDA), K-nearest Neighbors (KNN) and Ensemble classifier to differentiate the benign tumors from the malignant ones achieving an accuracy of 98.2%. Elmoufidi et al.^6^ proposed a method to segment and detect the boundary of different breast tissue regions in mammograms by using dynamic K-means clustering algorithm and Seed Based Region Growing (SBRG) techniques. Mammographic Image Analysis Society (MIAS) database is used for evaluation. Pratiwi et al^7^ proposed comparison of two classification methods: Radial Basis propagation Neural based on Gray-level Co-occurrence Matrix (GLCM) texture based features and BPNN. The computational experiments are performed on MIAS database showing that RBFNN is better than BPNN in breast cancer classification. Jen et al.^8^ proposed an efficient abnormality detection classifier (ADC) in mammogram images. Firstly, preprocessing is performed that included global equalization transformation, image denoising, binarization, breast object extraction, determination of breast orientation and the pectoral muscle suppression. On the obtained segmented images, gray level quantization is performed and further five features are extracted from the ROI and PCA is applied for determining feature weights. Azevedo et al.^9^ worked on IRMA database of mammograms that contains four types of tissues: fat, fibroid, dense and extremely dense. In this work, Morphological Extreme Learning machine is proposed with a hidden layer kernel based on morphological operators: dilation and erosion for classifying the masses as benign or malignant. Pereira et al.^3^ presents abnormality detection method in CC and MLO view of mammograms. Preprocessing included artifact removal algorithm followed by an image denoising and enhancement based on wavelet transform and Wiener filter. For segmentation of masses three techniques are used: multiple thresholding, wavelet transforms and genetic algorithm.DDSM database is used for experimentation Area overlap metric (AOM) achieved by the proposed method is 79%. Chen et al.^10^ proposed a clustering approach based on combination of fuzzy C-mean clustering (FCM) with PSO. As fuzzy C-mean clustering has some drawbacks such as the number of clusters needs to be specified in advance and also we need to have knowledge of the ground truth. The data points in overlapping areas cannot be correctly categorized so to overcome all these drawbacks PSO is used in combination with FCM. The result of this algorithm shows that it can automatically find the optimal number of clusters. Shanmugavadivu et al.^11^ proposed an intuitive segmentation technique to separate the microcalcification regions from the mammogram. This mechanism first enhances the input image using an image-dependent threshold value and binarizes it to obtain an enhanced image. Then the pixels constituting the edges of microcalcification regions are grown in the enhanced image, with respect to the neighbourhood pixels. Lastly, the edge intensities of the enhanced image are remapped into the original image, using which the regions of interest are segmented. The results of the present work are compared with the ground realities of the sample images obtained from MIAS database. Rao et al.^12^ proposed a new optimization method known as Teaching Learning- based optimization (TLBO) which is based on the influence of a teacher on the learners. It is a population-based method that uses a population of solution to reach to a global solution. Rangayyan et al.^13^ presents an overview of various digital image processing and pattern analysis techniques for problem solution in several areas of breast cancer diagnosis. This includes: contrast enhancement, detection and analysis of calcifications, masses and tumors, asymmetric shapes analysis and detection of architectural distortion. Mirjalili^14^ has utilized the GWO algorithm for training MLP for evaluating the accuracy and classification rate of three database issues. On the basis of experimental outcomes, proved that the GWO strategy is competent in giving the superior quality of outcomes in terms of enhanced local optima avoidance. After that the various authors have utilized the many recent enhanced and hybrid techniques on these database for effective solutions^15,16^.For locating the cancerous region in the mammogram image a comprehensive algorithm has been utilized by Sha^17^.This strategy has been applied on image noise reduction, optimal image segmentation for feature selection and extraction, thereby reducing the evaluating cost and enhancing precision etc. In addition, the proposed algorithm was utilized by the MIAS and DDSM databases. For verification, the performance of the presented approach has been validated with ten recent optimizers. Through tabulated outcomes it has been proved that the presented approach is competent to give the 96% Sensitivity, 93% Specificity, 85% PPV, 97% NPV, 92% accuracy, and better efficiency as compared to others. Shaikh et al.^18^ has introduced a new hybrid version by merging the features of harmony search (HS) and simulated annealing (SA) for precise and accurate breast malignancy. In this work, has been utilized 02 breast databases such as (i) benchmark BCDR-F03 database and (ii) local mammographic database. On the basis of tabulated outcomes have been proven the robustness of the proposed approach for these databases.

Houssein et al.^19^ gave a method to overcome the problem of abundant data analysis in cheminformatics.This paper proposed a hybrid method named CHHO-CS in which harris hawk optimizer is combined with two other operators i.e. cuckoo search and chaotic maps. Controlling of position vectors of HHO algorithm is done by cuckoo search. For prohibiting the control energy parameters to fall in local optima, chaotic maps are used. SVM is used as objective function alongwith CHHO-CS for identifying best features and removing repetitive data. This proposed method is tested on various databases and gave very promisisng results over traditional methods like HHO, moth-flame optimization, grey wolf optimizer and others. Houssein et al.^20^ proposed 3 modified versions named HHOCM i.e HHO with crossover and mutation CM, OBLHHOCM i.e opposite-based learning HHOCM and ROBLHHOCM i.e random opposition based learning HHOCM. These methods are tested on two datasets and results ensured best performance in finding subsets of molecular descriptors over other algorithms such as salp swarm optimization, original HHO, dragonfly algorithm, whale optimization algorithm in literature. Houssein et al.^21^ used Harris hawk optimization algorithm in large scale sensor network to find the sink node which helps the whole network in processing and analysis of information. As finding a sink node is a challenging task, HHO performed very well as compared to other optimization algorithms such as particle swarm optimization, sine cosine algorithm, flower pollination algorithms and others in literature. HHO gave good results in terms of increasing network lifetime and energy consumption for different size of networks as well as for multiple sink nodes. Recently a newly hybrid SCHHO approach has been developed by Hussain et al.^22^ for optimization problems and feature selection, which incorporates the features of sine-cosine algorithm (SCA) in Harris hawks optimization (HHO) algorithms for improving the exploration and exploitation performance. This approach is assessed through using recent test suites and sixteen datasets and compared with recent metaheuristics.

Nowadays the various hybrid and enhanced algorithms are developing by the researcher such as OHGBPPSO^23^, HMPSO^24^, HPSO^25^, MGWO^26^, MVGWO^27^, HSSAPSO^28^, SChoA^29^ and HSSASCA^30^ etc. After the inspiration of these algorithms have been developed the newly one hybrid algorithm during this work.

This proposed work presents by merging the features of dimension learning-based hunting (DLH) search strategy for database issues.In DLHO, the features of the DLH search strategy play an important role for maintaining diversity, ignoring the premature convergence and enhancing balance between exploitation and exploration phase of the HHO algorithm. Experimental outcomes have been compared and verified for the effectiveness of the proposed algorithm with the most recent optimizer strategies. To sum up, the main contributions of this new work are:

- A new enhanced version namely DLHO that includes features from DLH search strategy is proposed.
- DLHO is developed for solving 29-CEC-2017 test suites.
- To segment the cancerous region from mammogram, based on a fresh structure of the multilayer perceptron (MLP) neural network using DLHO algorithm.
- Utilizing DLHO algorithm for disease detection in other biomedical databases also.
- Statistical and qualitative experimental analyses to assess the robustness and effectiveness of the DLHO algorithm as compared to recent algorithms.

## Harris Hawks optimizer algorithm (HHO)

Harris Hawks optimizer approach is recently developed by Heidari et al.^31^. It’s a population based method and inspired through the intelligence of the crowd in which main inspiration is the co-operative behavior and chasing style of Harris’ hawks in nature called surprise pounce. During the hunting of the target each search agent or hawks jointly pounce prey by different positions. This methodology is implemented for finding the best target in the complex search space.

### Exploration stage

On the basis of the following equations, each search member randomly visits each location and waits to find a target;

1 $$\begin{aligned} X(t+1) = \left\{ \begin{matrix} X_{rand}(t)-r_{1}\left| X_{rand}(t)-2r_{2}X(t) \right| &{} q\ge 0.5 \\ (X_{rabbit}(t)-X_{m}(t))-r_{3}(LB+r_{4}(UB-LB)) &{} q<0.5 \end{matrix}\right. \end{aligned}$$

where $X(t+1)$ and *t*, $X_{rabbit}(t)$ are shows the the position vector of search agent and rabbit in the next generation, *X*(*t*) is show the recent location of search agents, $r_{1}$, $r_{2}$, $r_{3}$, $r_{4}$, and *q* are random numbers inside (0,1), which are updated in each iteration, *LB* and *UB* show the upper and lower bounds of variables, $X_{rand}(t)$ is a randomly selected search agent from the recent crowd, and $X_{m}$ is the average position of the recent crowd of search agents.

The mean location of the search member is attained by the following Eq. (2):

2 $$\begin{aligned} X_{m}(t)=\frac{1}{N}\sum _{i=1}^{N}X_{i}(t) \end{aligned}$$

where $X_{i}(t)$, *t* and *N* illustrates the position of each search agent, iteration and total number of search agents respectively.

#### Transition from exploration to exploitation

During this phase, energy of the prey is measured through the following Eq. 3;

3 $$\begin{aligned} E=2E_{0}(1-\frac{t}{T}) \end{aligned}$$

where *E*,*T* and $E_{0}$ illustrates the escaping energy of the target, total generations and initial state of its energy.

#### Exploitation phase

**Soft besiege** The behavior of the search agents is attained through the following conditions;

4 $$\begin{aligned}&X(t+1)=\Delta X(t)-E\left| JX_{rabbit}(t)-X(t)\right| \end{aligned}$$

5 $$\begin{aligned}&\Delta X(t)=X_{rabbit}(t)-X(t) \end{aligned}$$

where $J=2(1-r_{5})$, $\Delta X(t)$, $r_{5}$ and *t* are presents the random jump strength of the rabbit throughout the escaping procedure,the difference between the location vector of the rabbit, random number and recent position in generation.The *J* value changes randomly in all iterations to simulate the nature of prey or rabbit motions.

**Hard besiege** In this phase, the present positions of all agents are updated by Eq.(6):

6 $$\begin{aligned} X(t+1)=X_{rabbit}(t)-E \left| \Delta X(t) \right| \end{aligned}$$

**Soft besiege with progressive rapid dives**

The next move of the search member has been evaluated through the following (Eq. 7):

7 $$\begin{aligned} Y=X_{rabbit}(t)-E\left| JX_{rabbit}(t)-X(t)\right| \end{aligned}$$

Each member are drive by the following rule;

8 $$\begin{aligned} Z=Y+S\times LF(D) \end{aligned}$$

where *D*, $1\times D$ are presents the dimension of function, random vector and LF is the levy flight function, Which is calculated through the Eq. (9):

9 $$\begin{aligned} LF(x)=0.01\times \frac{u\times \sigma }{\left| v \right| ^{\frac{1}{\beta }}}, \sigma =\left( \frac{\Gamma (1+\beta )\times sin(\frac{\pi \beta }{2})}{\Gamma (\frac{1+\beta }{2})\times \beta \times 2^{(\frac{\beta -1}{2})})} \right) ^{\frac{1}{\beta }} \end{aligned}$$

where *u*, *v* and $\beta$ illustrates random values and default constant set to 1.5.

So, in this phase, for updating the positions of all members in the soft besiege stage can be performed by Eq. (10):

10 $$\begin{aligned} X(t+1)=\left\{ \begin{matrix} Y &{} if F(Y)<F(X(t)) \\ Z &{} if F(Z)<F(X(t)) \\ \end{matrix}\right. \end{aligned}$$

where *Y* and *Z* are obtained using Eqs. (7) and (8).

**Hard besiege with progressive rapid dives**

The following rule is performed in hard besiege condition:

11 $$\begin{aligned} X(t+1)=\left\{ \begin{matrix} Y &{} if F(Y)<F(X(t)) \\ Z &{} if F(Z)<F(X(t)) \\ \end{matrix}\right. \end{aligned}$$

where *Y* and *Z* are obtained using new rules in Eqs.(12) and (13).

12 $$\begin{aligned} Y=X_{rabbit}(t)-E\left| JX_{rabbit}(t)-X_{m}(t)\right| \end{aligned}$$

13 $$\begin{aligned} Z=Y+S\times LF(D) \end{aligned}$$

where $X_{m}(t)$ is obtained using Eq. (2).

## Enhanced DLHO Algorithm

The complex optimization applications are challenges for the optimization of meta-heuristics. According to literature, each optimization method is not able to show the best solution for all types of complex problems. All algorithms may face some drawbacks, so due to these weaknesses these could fail to find the solution of complex functions.

Cancer related issues are a big challenge for the bio-medical field researchers. Due to their complexity each optimization method is not competent to tackle these issues. Therefore, we always need the most robust optimizer method for the future demand. After this inspiration, to address these issues, a technique named DLHO has been developed by the merits of the HHO and dimension learning-based hunting (DLH) search strategy.In DLHO,the exploitation and exploration phases of HHO has been enhanced by DLH method to demonstrate the merits of DLH search strategy.DLH search strategy utilizes a dissimilar method to paradigm a neighborhood for each search member in which the neighboring information can be shared amid search agents.This strategy helps in maintaining the diversity and the balance amid global and local search. The exclusive motivation for overdue mixing modifications in HHO is to advantage the process to evade immature convergence and to steer the search in the way of possible exploration or search area in a faster direction.

By this mechanism, a hawk outcome is competent to escape from the local optima outcome. Also the accuracy or quality of the outcome is extended with faster convergence speed. With that the search members have explored the large search areas for trapping the superior outcome or goal. This procedure is repeated again and again until a new outcome or position has not fulfilled the termination conditions.

Mathematical phases of DLHO algorithm are as follows:

- **Parameters** The following parameter settings (see in Table 1) have been taken during the implementation of the algorithms.
- **Crowd initialization** Firstly, the crowd in the exploration area has been initialized randomly. In this, optimizers allocate a random $n_{d}$ for the $i^{th}$ hawk; $H_{i} (i=1,2,\ldots ,n)$. In the exploration area each slime has been allocated as:
  14 $$\begin{aligned} H= \begin{bmatrix} h_{1,1} &{} h_{1,2} &{}, \ldots , &{} h_{1,d}\\ h_{2,1} &{} h_{2,2} &{}, \ldots , &{} h_{2,d}\\ \vdots &{} \vdots &{} \ddots &{} \vdots \\ h_{n,1} &{} h_{n,2} &{}, \ldots , &{} h_{n,d}\\ \end{bmatrix} \end{aligned}$$
  Where *n* and *d* illustrate the hawks and size etc.
  The fitness of each slime has been amended by the equation (15);
  15 $$\begin{aligned} FH=\begin{bmatrix} fh_{1} \\ fh_{2} \\ \vdots \\ fh_{n} \\ \end{bmatrix} \end{aligned}$$
  where $fh_{i}$ illustrates the fitness outcome of the *i*th hawk during the searching procedure in the exploration space. In addition the following two distinct matrix can be formulated for target as in Eqs. ((16)–(17));
  16 $$\begin{aligned} T= & {} \begin{bmatrix} t_{1,1} &{} t_{1,2} &{}, \ldots , &{} t_{1,d}\\ t_{2,1} &{} t_{2,2} &{}, \ldots , &{} t_{2,d}\\ \vdots &{} \vdots &{} \ddots &{} \vdots \\ t_{n,1} &{} t_{n,2} &{}, \ldots , &{} t_{n,d}\\ \end{bmatrix} \end{aligned}$$
  17 $$\begin{aligned} FT= & {} \begin{bmatrix} ft_{1} \\ ft_{2} \\ \vdots \\ ft_{n} \\ \end{bmatrix} \end{aligned}$$
  where $ft_{i}$ denotes the fitness outcome for the final target.
- **Fitness Evaluation** The fitness value of the hawk is calculated through the following equations (18)-(19);
  18 $$\begin{aligned} F_{best}= & {} Min (fit_{j}(z)) \, \, j \varepsilon (1,2,\ldots ,n) \end{aligned}$$
  19 $$\begin{aligned} F_{worst}= & {} Max (fit_{j}(z)) \, \, j \varepsilon (1,2,\ldots ,n) \end{aligned}$$
  where $$F_{best}$$ and $$ F_{worst}$$ are illustrates the best and worst fitness value for functions.
- **Drive stage** During this phase, the DLH helps in specifically chasing the prey or goal of the search member in the search domain. DLH is used to construct a local area for each search member in which the best area or neighbors information can be shared between search members. Additionally, next phase represents how DLH and canonical HHO phases make two different search agents.
- **Canonical HHO phase** In HHO method phase, the position of the all member in the search domain is updated by equations ((1)–(13)).Finally the first search member for the new location or position of HHO $X_i(t)$ named $X_{HHO} (t+1)$ is updated by equation (11).
- **DLH phase** In DLH, the position of the search agents is modified by an equation in which this different search agent is learned by its different neighbors or local optima and a randomly chosen search member from the population.After that, besides $X_{i-HHO} (t+1)$, the DLH phase creates other candidate for the new position of hawk $X_{i}(t)$ named $X_{i-DLH}(t+1)$. To do this, a radius $R_{t}(n)$ is evaluated applying Euclidean distance among present location of hawk $X_{i}(t)$ and the candidate position $X_{i-HHO}$ by equation (20).
  20 $$\begin{aligned} R_{i}(n)=\left\| X_{i}(t)-X_{i-HHO}(t+1) \right\| \end{aligned}$$
  Then, the near hawks of $X_{i}(t)$ indicated by $L_{t}(n)$ are calculated by equation (21) with respect to equation (20).Here $M_{i}$ illustrates the Euclidean distance amid $X_{i}$ and $X_{i}$.
  21 $$\begin{aligned} L_{i}(t)=\left\{ X_{i}(t),M_{i}( X_{i}(t),X_{j}(t))\le R_{i}(t),X_{j}(t)\epsilon \,crowd\right\} \end{aligned}$$
  After that, the multi-neighbors learning is executed by the following mathematical formulation of equation (22);
  22 $$\begin{aligned} X_{i-DLH,d}(t+1)=X_{i,d}(t)+rand \times (X_{m,d}(t)-x_{r,d}(t)) \end{aligned}$$
  where *d* is illustrate the dimension.
- **Position update stage** In this phase, the best hawk is nominated by comparing the fitness outputs of two hawks $X_{i-HHO}(t+1)$ and $X_{i-DLH}(t+1)$ by equation;
  23 $$\begin{aligned} X_{i}(t+1)=\left\{ \begin{matrix} X_{i-HHO}(t+1) &{} if \, \, f(x_{i-HHO})< f(X_{i-DLH})\\ X_{i-DLH}(t+1) &{} otherwise \end{matrix} \right. \end{aligned}$$
  So, as per above equation if the fitness output of the chosen member is $< X_{i}(t)$, then $X_{i}(t)$ is modified by the chosen member. Otherwise, $X_{i}(t)$ remains unchanged in the crowd.
- **Stopping Condition** Under this stage, the stopping criteria are utilized for modifying position of the hawks in the search domain. This process is repetitive again and again, until it does not fulfill the conditions of prevention.

**Table 1:** Parameter settings for algorithms.

-	MFO	SCA	Chimp	SBPO	AOA	SMA	DLHO
Parameters	.	.	Values	.	.	.	
a	–1	–	–	–	–	–	–
b	1	–	–	–	–	–	–
Max.iter	500	500	500	500	500	500	500
Crowd size	30	30	30	30	30	30	30
lb	10	10	10	10	10	10	10
ub	100	100	100	100	100	100	100
No. of (runs)	30	30	30	30	30	30	30
Max NFFE	–	–	–	10E+5	–	–	–

### Pseudocode of DLHO

The pseudocode of DLHO algorithm is reported in Algorithm 1.

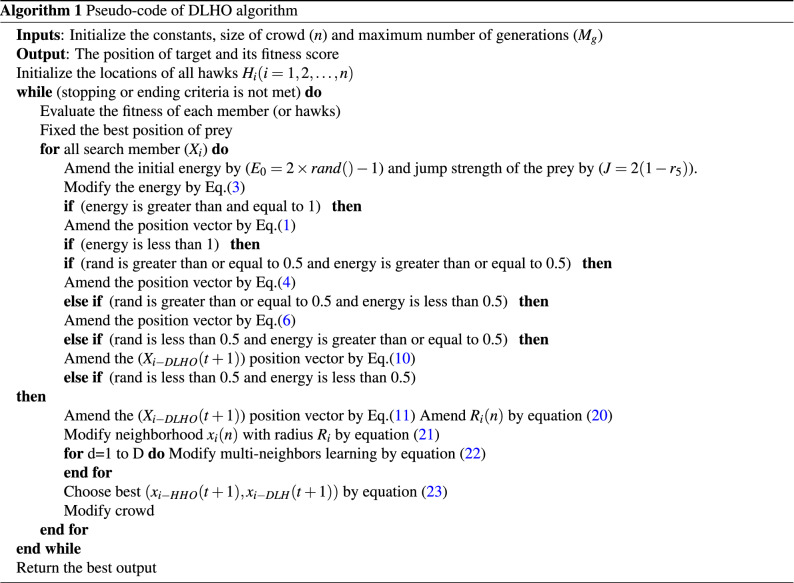



## Analysis and discussions

### Analysis

The robustness of the new method has been evaluated on 29-CEC- test suite and verified by the recent methods such as MFO^32^, SCA^33^, Chimp^34^, SBPSO^35^ and AOA^36^, SMA^37^ etc. In addition, the accuracy, robustness and effectiveness^38^ of the new technique are discussed in following subsections:

#### Test suites and constants

All methods have been coded in R-Matlab-2018a software and tested on 8GB Ram with 64 for bit operating system and Core i3, 8th Gen system for evaluating the robustness performance of the algorithms. Under this implementation of these methods the various constant settings have been fixed such as search agents ($n=30$), size of problems (10–100), upper and lower size taken amind –100 to 100 respectively.

**Figure 2:**
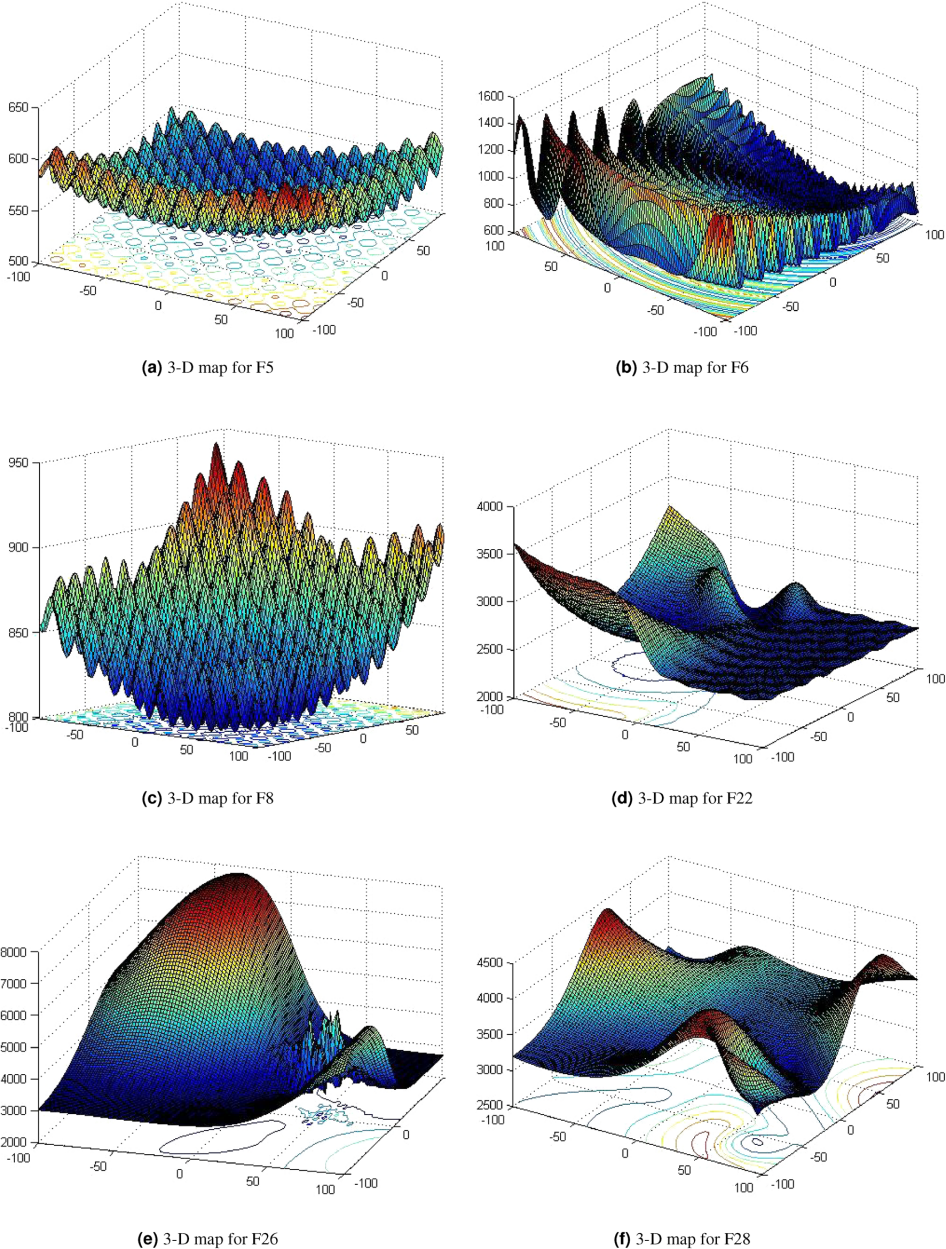
3-D graphs of CEC’2017 test suites

The performance of the proposed method has been tested on 29 CEC functions, these shown in Table 2^39^. The three dimension graphs of CEC are illustrated by Fig. 2. Normally, these suites could be divided into four phases as uni-modal, simple multi-modal, hybrid and composition etc.

**Table 2:** Summary of the CEC’2017 test suite.

Name	No.	Function	$F^*_i=F(x^*)$
Unimodal	f1	Shifted and Rotated Bent Cigar Function	100
Unimodal	f2	Shifted and Rotated Zakharov Function	200
Simple Multimodal	f3	Shifted and Rotated Rosenbrock’s Function	300
Simple Multimodal	f4	Shifted and Rotated Rastrigin’s Function	400
Simple Multimodal	f5	Shifted and Rotated Expanded Scaffer’s F6 Function	500
Simple Multimodal	f6	Shifted and Rotated Lunacek Bi-Rastrigin Function	600
Simple Multimodal	f7	Shifted and Rotated Non-Continuous Rastrigin’s Function	700
Simple Multimodal	f8	Shifted and Rotated Levy Function	800
Simple Multimodal	f9	Shifted and Rotated Schwefel’s Function	900
Hybrid	f10	Hybrid Function 1 (N=3)	1000
Hybrid	f11	Hybrid Function 2 (N=3)	1100
Hybrid	f12	Hybrid Function 3 (N=3)	1200
Hybrid	f13	Hybrid Function 4 (N=4)	1300
Hybrid	f14	Hybrid Function 5 (N=4)	1400
Hybrid	f15	Hybrid Function 6 (N=4)	1500
Hybrid	f16	Hybrid Function 6 (N=5)	1600
Hybrid	f17	Hybrid Function 6 (N=5)	1700
Hybrid	f18	Hybrid Function 6 (N=5)	1800
Hybrid	f19	Hybrid Function 6 (N=6)	1900
Composition	f20	Composition Function 1 (N=3)	2000
Composition	f21	Composition Function 2 (N=3)	2100
Composition	f22	Composition Function 3 (N=4)	2200
Composition	f23	Composition Function 4 (N=4)	2300
Composition	f24	Composition Function 5 (N=5)	2400
Composition	f25	Composition Function 6 (N=5)	2500
Composition	f26	Composition Function 7 (N=6)	2600
Composition	f27	Composition Function 8 (N=6)	2700
Composition	f28	Composition Function 9 (N=3)	2800
Composition	f29	Composition Function 10 (N=3)	2900
-	-	search range [-100,100]$^D$	-

#### Testing and evaluation

To confirm the effectiveness, robustness and accuracy of the proposed optimizer it has been run on 29-CEC test suite. Numerical and statistical results of the proposed optimizer proves that the proposed method is able to give the highly effective and accurate solutions in terms of min and max objective scores, mean and standard scores etc. Results of the optimizers are denoted by Tables 3–6 and Figs. 4–7.

Further, in the following subsections, the brief details of the results analysis and discussions have been described.

### Discussions on the experimental results

Generally the test suites can be split into four phases and each phase is utilized to prove the robustness of the optimizer on different levels such as;

- **Uni-modal:** These are utilized to present the exploitation capability of the optimizers.
- **Multi-modal:** These functions involves various local optima that are applied to examine the capability of optimizers for many optima.
- **Hybrid and Composite:** These functions are used to assess the exploration capability of the optimizers.

#### Exploitation competence

Uni-modal functions involve a single global optima normally used for evaluating the exploitation performance of the optimizers. Results in Tables (3–5), illustrated that the DLHO is competent to give the best exploitation performance in the search space as compared to other optimizers. Experiments have proven that the DLHO method can handle the uni-modal functions easily and is able to give the best and accurate score on these functions than others. Here, it could be concluded that the enhancement of HHO method allowed the functions to reach the best global optima. So, the DLHO method could tackle the high domain and complex functions easily.

As specified previously, the CEC test suites are more suitable functions for evaluating or testing the robustness of the optimizers. All Simulation proves that the DLHO is extremely functional. Results give strong evidence that the DLHO is competent to give the most effective and accurate optima solutions for the complex domain functions.

#### Competence valuation

Multi-modal functions are more suitable for testing the suitability of the optimizer since these involve many local optima, and the number of variables exceed exponentially against the size of the function as compared to the uni-modal function. Results show that the proposed method shows strong detection behavior.

**Table 3:** Results of Optimizers on Uni-modal, Simple Multi-modal test suites.

F	MFO		SCA		Chimp		SBPO	
f1-29	$F_{min}$	$F_{max}$	$F_{min}$	$F_{max}$	$F_{min}$	$F_{max}$	$F_{min}$	$F_{max}$
f1	1.03E+09	1.30E+11	1.68E+10	1.26E+11	2.44E+10	1.14E+11	6.03E+10	9.65E+10
f2	3.03E+03	2.36E+06	2.19E+03	2.17E+05	5.55E+03	1.03E+06	3.16E+04	3.16E+04
f3	9.99E+02	4.36E+04	2.65E+03	3.64E+04	5.42E+03	4.59E+04	6.54E+03	2.69E+04
f4	7.58E+02	1.12E+03	8.40E+02	1.24E+03	8.45E+02	1.20E+03	9.39E+02	1.07E+03
f5	6.20E+02	7.38E+02	6.73E+02	7.21E+02	6.71E+02	7.34E+02	6.90E+02	7.32E+02
f6	9.00E+02	3.67E+03	1.29E+03	3.98E+03	1.20E+03	3.55E+03	3.02E+03	3.06E+03
f7	9.85E+02	1.40E+03	1.13E+03	1.36E+03	1.12E+03	1.34E+03	1.23E+03	1.29E+03
f8	6.16E+03	4.33E+04	7.79E+03	4.66E+04	7.71E+03	4.63E+04	1.79E+04	1.87E+04
f9	6.29E+03	1.10E+04	8.75E+03	9.89E+03	8.31E+03	1.17E+04	7.31E+03	7.96E+03
f10	1.33E+03	1.05E+04	1.19E+03	2.16E+03	1.49E+03	9.47E+03	4.36E+04	8.86E+04
f11	2.75E+08	3.02E+10	1.33E+09	2.04E+10	4.75E+09	2.14E+10	8.90E+09	8.90E+09
f12	4.24E+05	4.99E+10	1.71E+09	2.14E+10	4.23E+09	2.93E+10	8.91E+09	3.66E+10
f13	2.73E+05	2.37E+07	1.83E+05	2.50E+07	2.27E+06	1.76E+07	1.85E+07	1.85E+07
f14	1.18E+09	8.07E+09	4.52E+07	1.30E+10	2.13E+07	7.38E+09	7.79E+09	7.79E+09
f15	3.20E+03	1.83E+04	3.72E+03	1.09E+04	4.61E+03	7.86E+03	5.28E+03	6.25E+03
f16	2.52E+03	1.16E+05	2.53E+03	3.53E+04	2.92E+03	4.15E+04	3.29E+03	8.08E+03
f17	1.18E+06	1.95E+09	5.84E+06	1.14E+09	6.01E+06	1.15E+09	1.30E+08	1.32E+08
f18	6.22E+04	1.30E+10	1.61E+08	9.39E+09	3.93E+06	1.14E+10	8.44E+09	1.44E+10
f19	2.70E+03	3.70E+03	2.96E+03	4.30E+03	3.34E+03	4.03E+03	3.25E+03	3.51E+03
f20	2.46E+03	2.99E+03	2.61E+03	2.96E+03	2.63E+03	2.92E+03	2.71E+03	2.76E+03
f21	7.13E+03	1.24E+04	1.02E+04	1.27E+04	1.00E+04	1.25E+04	8.72E+03	1.00E+04
f22	2.62E+03	2.79E+03	2.67E+03	2.78E+03	2.67E+03	2.86E+03	2.69E+03	2.72E+03
f23	2.98E+03	4.53E+03	3.31E+03	4.07E+03	3.33E+03	4.20E+03	3.23E+03	3.33E+03
f24	4.89E+03	1.92E+04	3.32E+03	1.31E+04	5.35E+03	1.73E+04	8.48E+03	1.34E+04
f25	5.80E+03	2.40E+04	7.59E+03	1.31E+04	7.54E+03	1.55E+04	9.18E+03	1.03E+04
f26	3.75E+03	9.49E+03	4.84E+03	8.33E+03	5.02E+03	9.75E+03	3.96E+03	4.26E+03
f27	6.89E+03	2.76E+04	9.16E+03	2.91E+04	7.45E+03	1.98E+04	1.08E+04	1.38E+04
f28	3.26E+03	4.26E+03	3.34E+03	3.73E+03	3.45E+03	3.90E+03	3.46E+03	3.87E+03
f29	7.88E+05	3.54E+09	2.30E+08	4.72E+09	1.39E+08	2.74E+09	2.25E+09	3.04E+09

#### Balance Competence valuation

In hybrid and composite functions various local and global optima are involved normally which are utilized for evaluating the exploration robustness of the optimizers. In addition, these suites are utilized for evaluating and verifying the balance amid exploration and exploitation phases of the optimizers. Results showed that the DLHO is competent to make a strong balance amid exploration and exploitation phases. So, by this enhancement we can find the most effective solutions for the complex functions which illustrate the strong exploitation and exploration behavior of the DLHO method. Furthermore, this modification helps to make the new position or other best fitness location for each search agent which helps to amend their present locations. Experimental measurements illustrate the effective detection behavior of the DLHO for trapping the best scores against the least number of generations. Since hybrid and composite suites involve more complex space which shows the robustness of the new method. Hence these experiments showed that the proposed method leads to best optima by the most effective scan behavior.

Additionally, the DLHO involves robust local and global optima prevention which helps in trapping the best goal quickly.

**Table 4:** Results of Optimizers on Hybrid and Composite test suites.

F	AOA		SMA		DLHO	
f1-29	$F_{min}$	$F_{max}$	$F_{min}$	$F_{max}$	$F_{min}$	$F_{max}$
f1	4.69E+10	1.18E+10	1.05E+05	1.15E+11	**3.02E+04**	**1.66E+11**
f2	2.50E+03	1.50E+05	2.21E+04	3.22E+05	**2.77E+02**	**7.39E+06**
f3	9.96E+03	4.36E+04	5.44E+02	2.81E+04	**3.23E+02**	**6.65E+04**
f4	8.71E+02	1.19E+03	6.55E+02	1.24E+03	**6.12E+02**	**2.01E+03**
f5	6.76E+02	7.56E+02	6.25E+02	7.30E+02	**5.24E+02**	**7.98E+02**
f6	1.40E+03	3.54E+03	9.02E+02	3.73E+03	**6.88E+02**	**5.34E+03**
f7	1.13E+03	1.41E+03	9.39E+02	1.43E+03	**8.45E+02**	**1.43E+03**
f8	1.79E+04	1.98E+04	3.27E+03	4.11E+04	**1.12E+03**	**5.66E+04**
f9	8.69E+03	1.04E+04	5.07E+03	1.16E+04	**3.76E+03**	**2.67E+04**
f10	1.52E+03	6.21E+03	1.34E+03	2.28E+04	**1.05E+03**	**9.99E+04**
f11	1.7780+10	4.06E+10	1.08E+07	2.16E+10	**1.55E+06**	**5.03E+10**
f12	5.60E+09	2.37E+10	5.71E+04	3.75E+10	**2.09E+04**	**6.05E+10**
f13	2.27E+06	2.39E+07	2.40E+05	2.54E+07	**1.02E+05**	**4.88E+07**
f14	1.29E+09	1.04E+10	4.63E+04	8.44E+09	**1.34E+03**	**2.12E+10**
f15	5.60E+03	1.80E+04	3.46E+03	9.50E+03	**2.22E+03**	**5.09E+04**
f16	3.02E+03	4.23E+03	2.37E+03	3.72E+04	**1.99E+03**	**4.09E+04**
f17	2.29E+07	1.36E+09	3.89E+06	1.48E+09	**3.77E+05**	**2.88E+09**
f18	2.76E+09	1.92E+10	2.28E+04	1.62E+10	**1.04E+04**	**2.58E+10**
f19	2.76E+03	3.85E+03	2.52E+03	3.85E+03	**1.90E+03**	**5.12E+03**
f20	2.63E+03	2.91E+03	2.44E+03	2.98E+03	**1.87E+03**	**4.01E+03**
f21	1.01E+04	1.25E+04	7.41E+03	1.21E+04	**4.08E+03**	**2.12E+04**
f22	2.67E+03	2.84E+03	2.63E+03	2.92E+03	**1.09E+03**	**3.33E+03**
f23	3.98E+03	4.80E+03	2.96E+03	4.32E+03	**2.05E+03**	**5.07E+03**
f24	4.27E+03	2.57E+04	7.21E+03	1.66E+04	**1.79E+03**	**3.16E+04**
f25	1.02E+04	1.48E+04	5.78E+03	1.77E+04	**4.44E+03**	**3.34E+04**
f26	8.01E+03	1.15E+04	3.57E+03	8.31E+03	**1.87E+03**	**2.77E+04**
f27	1.23E+04	2.48E+04	1.27E+04	3.22E+04	**2.11E+03**	**4.96E+04**
f28	3.31E+03	4.18E+03	3.27E+03	4.54E+03	**2.88E+03**	**4.85E+03**
f29	1.61E+09	5.04E+09	1.74E+05	3.15E+09	**1.87E+04**	**4.01E+09**

**Table 5:** Mean ($\mu$) outcomes of Optimizers on the 29-CEC-2017 functions.

F	MFO	SCA	Chimp	SBPO	AOA	SMA	DLHO
F1-23	$\mu$	$\mu$	$\mu$	$\mu$	$\mu$	$\mu$	$\mu$
f1	1.61E+10	3.57E+10	8.21E+10	6.27E+10	4.74E+10	1.42E+10	**4.88E+09**
f2	1.97E+04	2.07E+04	3.71E+04	3.16E+04	4.88E+03	3.73E+04	**4.12E+03**
f3	3.24E+03	9.78E+03	3.03E+04	7.31E+03	1.04E+04	3.05E+03	**1.01E+03**
f4	7.95E+02	9.47E+02	1.04E+03	9.41E+02	8.77E+02	7.85E+02	**6.77E+02**
f5	6.38E+02	6.91E+02	7.10E+02	6.90E+02	6.79E+02	6.58E+02	**5.66E+02**
f6	1.33E+03	1.88E+03	2.35E+03	3.02E+03	1.42E+03	1.14E+03	**1.00E+03**
f7	1.04E+03	1.18E+03	1.24E+03	1.23E+03	1.14E+03	1.04E+03	**8.77E+02**
f8	9.09E+03	1.71E+04	3.03E+04	1.79E+04	1.11E+04	1.79E+04	**5.99E+03**
f9	7.00E+03	9.19E+03	8.87E+03	7.33E+03	8.97E+03	7.46E+03	**5.01E+03**
f10	1.59E+03	1.51E+03	2.44E+03	4.48E+04	1.92E+03	1.84E+03	**1.21E+03**
f11	1.50E+09	5.74E+09	1.25E+10	5.67E+09	1.81E+10	1.72E+09	**6.43E+08**
f12	1.00E+09	4.51E+09	1.16E+10	9.06E+09	6.28E+09	9.83E+08	**3.22E+08**
f13	9.72E+05	3.94E+06	1.20E+07	1.85E+07	3.35E+06	1.42E+06	**8.17E+05**
f14	1.23E+09	6.46E+08	1.41E+09	7.79E+09	1.36E+09	3.27E+08	**1.02E+08**
f15	3.54E+03	4.34E+03	6.24E+03	5.49E+03	5.72E+03	4.10E+03	**1.88E+03**
f16	3.23E+03	3.45E+03	4.21E+03	3.39E+03	3.04E+03	2.95E+03	**1.97E+03**
f17	2.13E+07	4.81E+07	4.86E+07	1.30E+08	3.26E+07	4.11E+07	**1.02E+07**
f18	2.49E+08	6.37E+08	2.56E+09	9.80E+09	2.87E+09	6.37E+08	**1.13E+08**
f19	2.77E+03	3.17E+03	3.55E+03	3.26E+03	3.03E+03	2.79E+03	**1.98E+03**
f20	2.53E+03	2.68E+03	2.79E+03	2.71E+03	2.64E+03	2.54E+03	**1.67E+03**
f21	7.73E+03	1.05E+04	1.04E+04	8.77E+03	1.06E+04	9.48E+03	**4.16E+03**
f22	2.63E+03	2.67E+03	2.69E+03	2.69E+03	2.67E+03	2.64E+03	**1.66E+03**
f23	3.05E+03	3.35E+03	3.52E+03	3.23E+03	3.99E+03	3.06E+03	**1.88E+03**
f24	6.06E+03	5.61E+03	1.16E+04	8.66E+03	4.41E+03	7.27E+03	**1.49E+03**
f25	6.68E+03	8.63E+03	1.01E+04	9.21E+03	1.03E+04	6.87E+03	**4.22E+03**
f26	3.92E+03	5.07E+03	6.76E+03	3.97E+03	8.26E+03	3.87E+03	**1.04E+03**
f27	9.89E+03	1.36E+04	1.39E+04	1.09E+04	1.08E+04	1.67E+04	**3.44E+03**
f28	3.28E+03	3.42E+03	3.59E+03	3.47E+03	3.33E+03	3.31E+03	**2.59E+03**
f29	6.93E+07	3.85E+08	7.27E+08	2.26E+09	1.64E+09	1.90E+08	**3.98E+07**

**Table 6:** Standard deviation (*sd*) outcomes of optimizers on 29-CEC-2017 functions.

F	MFO	SCA	Chimp	SBPO	AOA	SMA	DLHO
F1-23	*sd*	*sd*	*sd*	*sd*	*sd*	*sd*	*sd*
f1	2.63E+10	2.44E+10	5.36E+10	6.06E+09	4.15E+09	3.04E+10	**1.22E+09**
f2	1.49E+05	3.07E+04	6.54E+04	5.83E+04	1.82E+04	5.26E+04	**1.02E+04**
f3	6.64E+03	8.36E+03	2.55E+04	2.40E+03	3.20E+03	6.01E+03	**1.32E+03**
f4	6.99E+01	1.14E+02	1.57E+02	1.12E+01	2.86E+01	1.40E+02	**7.55E+00**
f5	2.64E+01	3.77E+01	2.79E+01	2.25E+00	5.71E+00	2.94E+01	**1.17E+00**
f6	3.87E+02	8.77E+02	1.00E+03	7.12E+00	1.23E+02	2.84E+02	**2.98E+00**
f7	7.66E+02	7.77E+01	9.85E+01	2.92E+00	2.43E+01	1.02E+02	**1.44E+00**
f8	5.77E+03	9.16E+03	1.54E+04	3.44E+01	2.08E+03	6.61E+03	**1.98E+01**
f9	1.21E+03	5.08E+02	5.83E+02	1.01E+02	2.86E+02	1.78E+03	**1.20E+01**
f10	9.29E+02	3.73E+02	1.77E+03	5.49E+03	1.30E+03	2.62E+03	**0.98E+03**
f11	4.07E+09	5.29E+09	6.85E+09	6.34E+09	1.82E+09	4.79E+09	**2.87E+00**
f12	4.93E+09	4.47E+09	9.20E+09	1.72E+09	1.64E+09	3.21E+09	**1.00E+09**
f13	3.07E+06	6.95E+06	2.29E+07	4.77E+06	3.51E+06	3.19E+06	**1.23E+06**
f14	4.51E+08	1.04E+09	2.41E+09	1.68E+09	5.47E+08	1.05E+09	**2.20E+08**
f15	1.26E+03	7.93E+02	1.24E+03	2.66E+02	7.06E+02	8.31E+02	**0.81E+02**
f16	7.37E+03	2.10E+03	2.21E+03	3.55E+02	3.01E+02	2.71E+03	**1.76E+02**
f17	1.33E+08	8.13E+07	1.26E+08	7.70E+07	8.46E+07	1.85E+08	**3.44E+07**
f18	1.35E+09	1.21E+09	4.42E+09	2.51E+09	9.44E+08	1.32E+09	**0.66E+09**
f19	1.83E+02	2.40E+02	2.03E+02	2.96E+01	2.92E+02	3.05E+02	**1.33E+01**
f20	1.19E+02	1.29E+02	1.20E+02	7.43E+00	3.05E+01	1.33E+02	**1.19E+00**
f21	1.14E+03	6.90E+02	3.93E+02	1.85E+02	2.94E+02	1.44E+03	**3.14E+01**
f22	2.27E+01	1.20E+02	2.38E+01	7.65E+00	2.11E+01	2.22E+01	**2.30E+00**
f23	1.66E+02	1.74E+02	1.93E+02	5.44E+00	7.15E+01	1.32E+02	**2.64E+00**
f24	2.76E+03	3.11E+03	5.31E+03	8.01E+02	1.26E+03	7.25E+02	**1.01E+02**
f25	2.16E+03	1.44E+03	2.51E+03	1.31E+02	4.24E+02	1.66E+03	**4.90E+01**
f26	6.29E+02	7.06E+02	1.72E+03	6.78E+02	6.66E+02	6.28E+02	**1.21E+02**
f27	3.68E+03	3.99E+03	5.37E+03	2.33E+02	1.62E+03	2.74E+03	**1.00E+02**
f28	1.06E+02	1.73E+02	1.45E+02	4.31E+01	5.30E+01	1.19E+02	**1.54E+01**
f29	3.73E+08	4.39E+08	6.96E+08	4.24E+07	1.81E+08	6.08E+08	**2.08E+07**

#### Accuracy

In this phase the accuracy behavior of the proposed optimizer will be discussed. In general, the least average score shows the accuracy of the optimizer against the best score. The best average scores of the optimizer are illustrated in a Table 7 into two different phases such as best (B) and worst (W) respectively. The best and worst average scores have been assigned in Table 7 by the outcomes of the Table 5. Results of the table show that the DLHO is competent to trap the best score against the least average score than others. Hence it can be said that the new enhanced version is competent to trap the best and accurate outcomes for the complex issues as comparison to others.

**Table 7:** Best and worst mean ($\mu$) outcomes of optimizers on 29-CEC-2017 functions.

F	MFO	SCA	Chimp	SBPO	AOA	SMA	DLHO
f1	W	W	W	W	W	W	B
f2	W	W	W	W	W	W	B
f3	W	W	W	W	W	W	B
f4	W	W	W	W	W	W	B
f5	W	W	W	W	W	W	B
f6	W	W	W	W	W	W	B
f7	W	W	W	W	W	W	B
f8	W	W	W	W	W	W	B
f9	W	W	W	W	W	W	B
f10	W	W	W	W	W	W	B
f11	W	W	W	W	W	W	B
f12	W	W	W	W	W	W	B
f13	W	W	W	W	W	W	B
f14	W	W	W	W	W	W	B
f15	W	W	W	W	W	W	B
f16	W	W	W	W	W	W	B
f17	W	W	W	W	W	W	B
f18	W	W	W	W	W	W	B
f19	W	W	W	W	W	W	B
f20	W	W	W	W	W	W	B
f21	W	W	W	W	W	W	B
f22	W	W	W	W	W	W	B
f23	W	W	W	W	W	W	B
f24	W	W	W	W	W	W	B
f25	W	W	W	W	W	W	B
f26	W	W	W	W	W	W	B
f27	W	W	W	W	W	W	B
f28	W	W	W	W	W	W	B
f29	W	W	W	W	W	W	B

#### Stability

**Figure 3:**
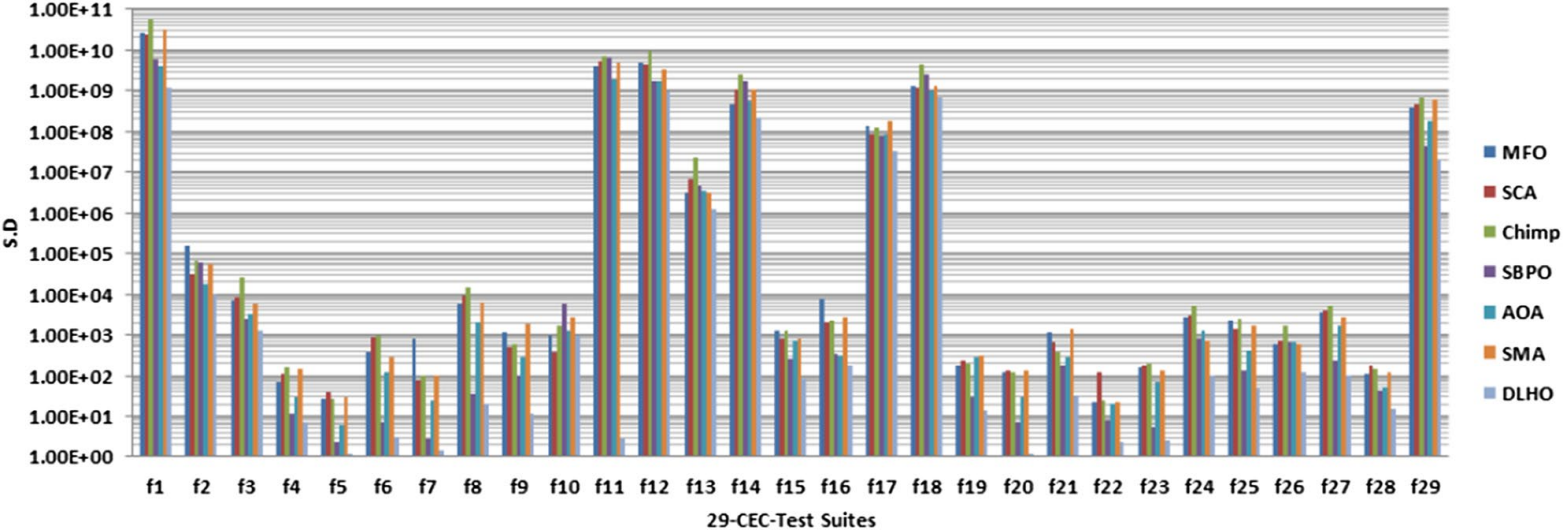
Statistical best (*sd*) solutions graph of algorithms on the 29-CEC’2017 suites.

In general, if the standard scores lie near to ’0’ then these illustrates the stability of the optimizers for the global optima in the search space. The standard scores of the optimizers have been plotted by the Fig. 3 against the Table 6. In Fig. 3, it can be easily seen that the DLHO method is finding the best global optima for each function against the least standard score than others. These results show better stability performance of the new version than others on all functions. Additionally the least *sd* score also illustrates the convergence speed of the optimizers. On the basis of the all outcomes of Table 6 and Fig. 3 it can be concluded that the DLHO is competent to finding the best outcomes for complex issues much more fast without losing their track.

#### Convergence graphs analysis and discussion

**Figure 4:**
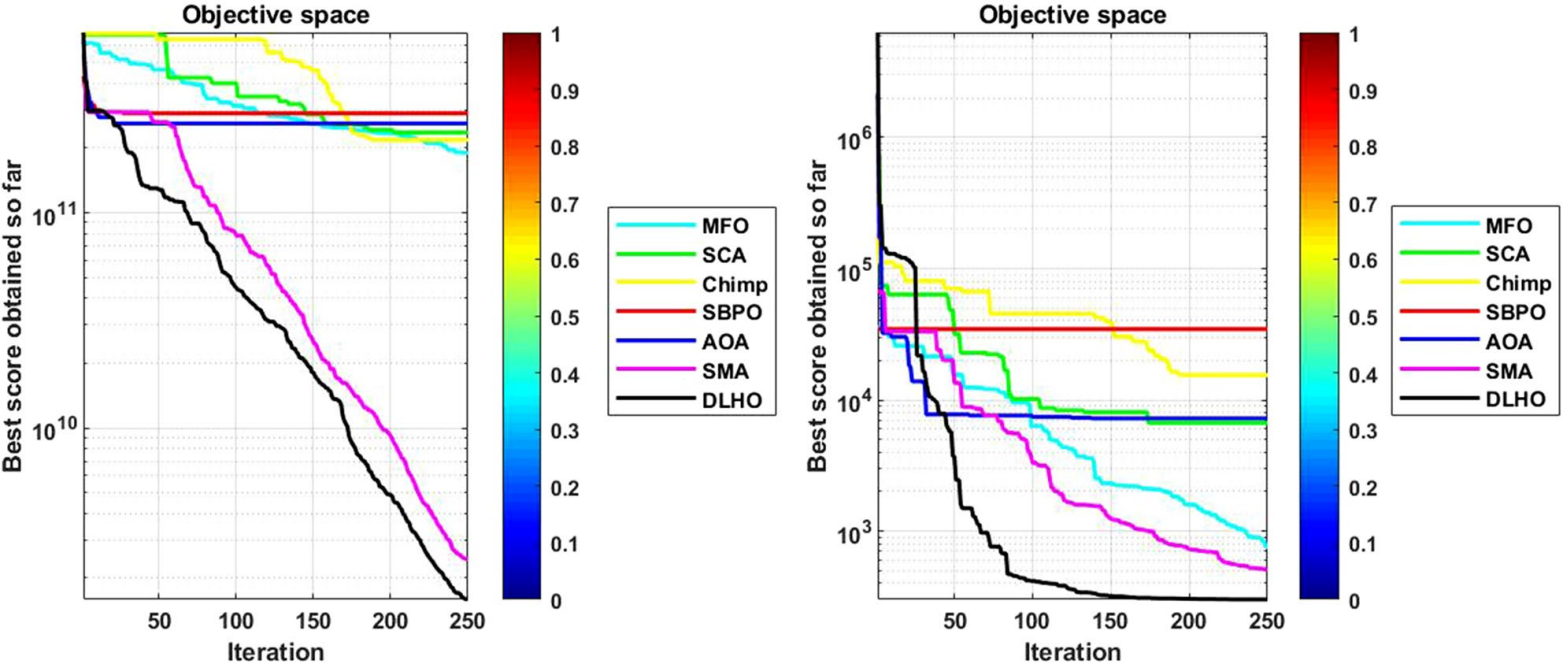
Performance graphs of Optimizers on uni-modal test suites.

**Figure 5:**
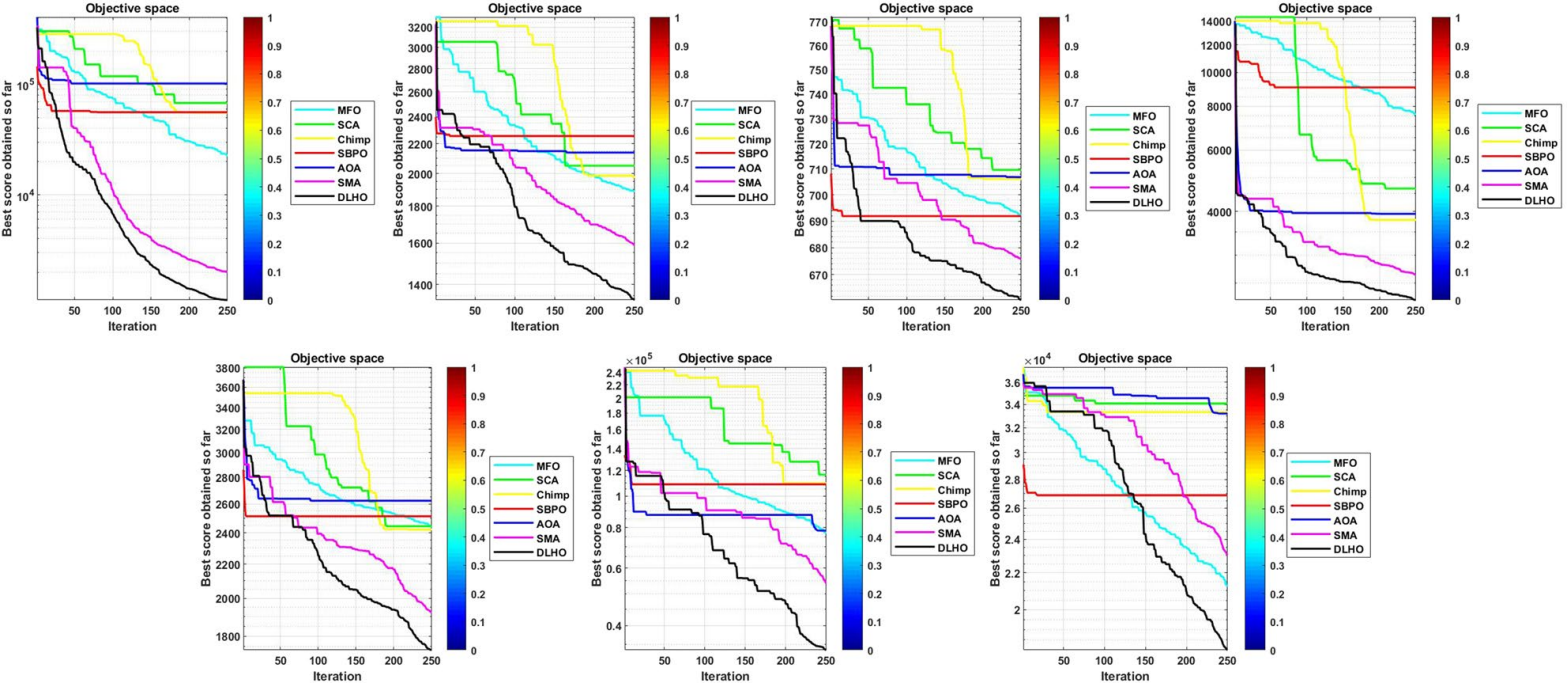
Performance graphs of Optimizers on simple-multimodal test suites.

**Figure 6:**
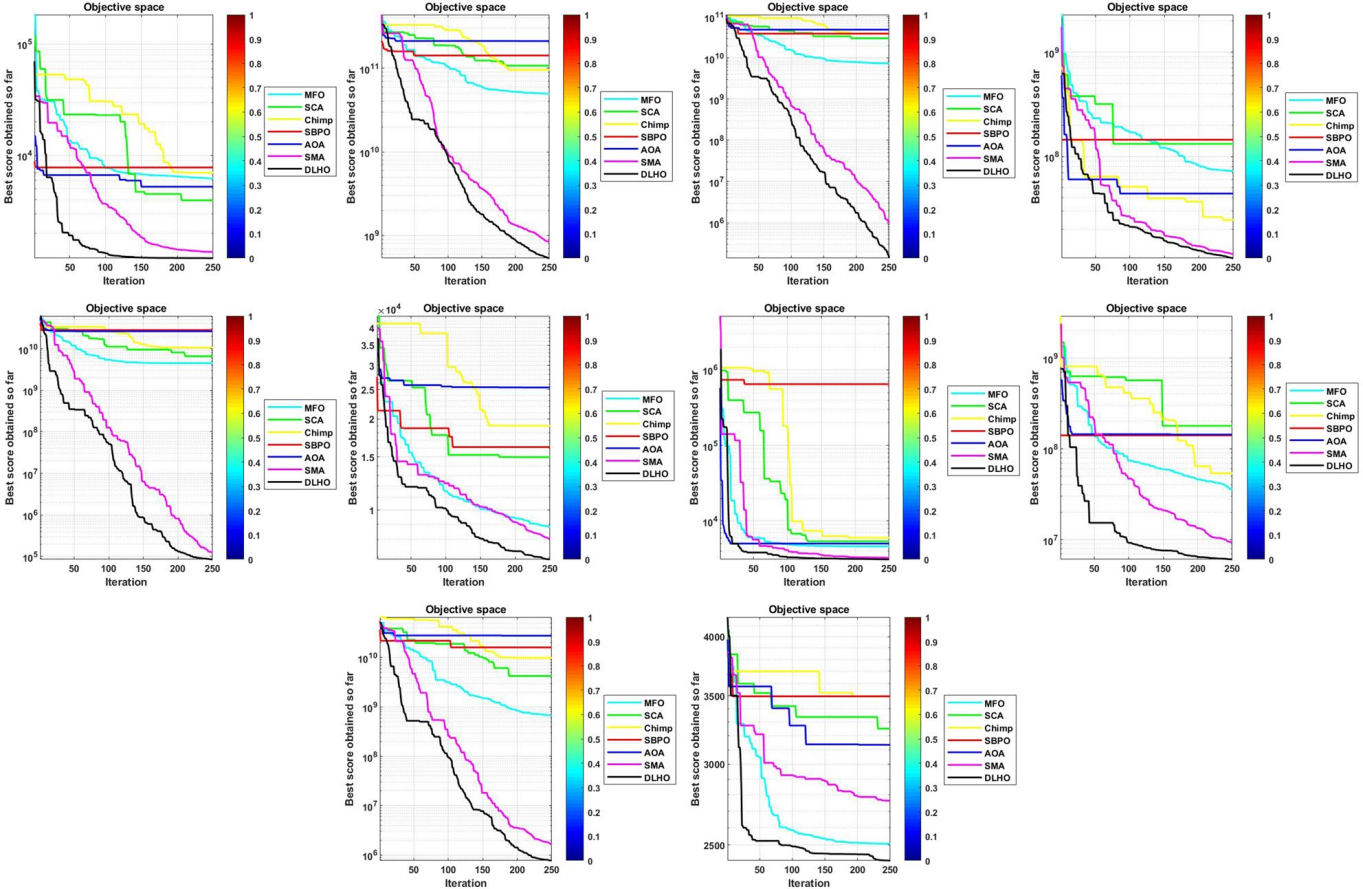
Performance graphs of Optimizers on hybrid test suites.

**Figure 7:**
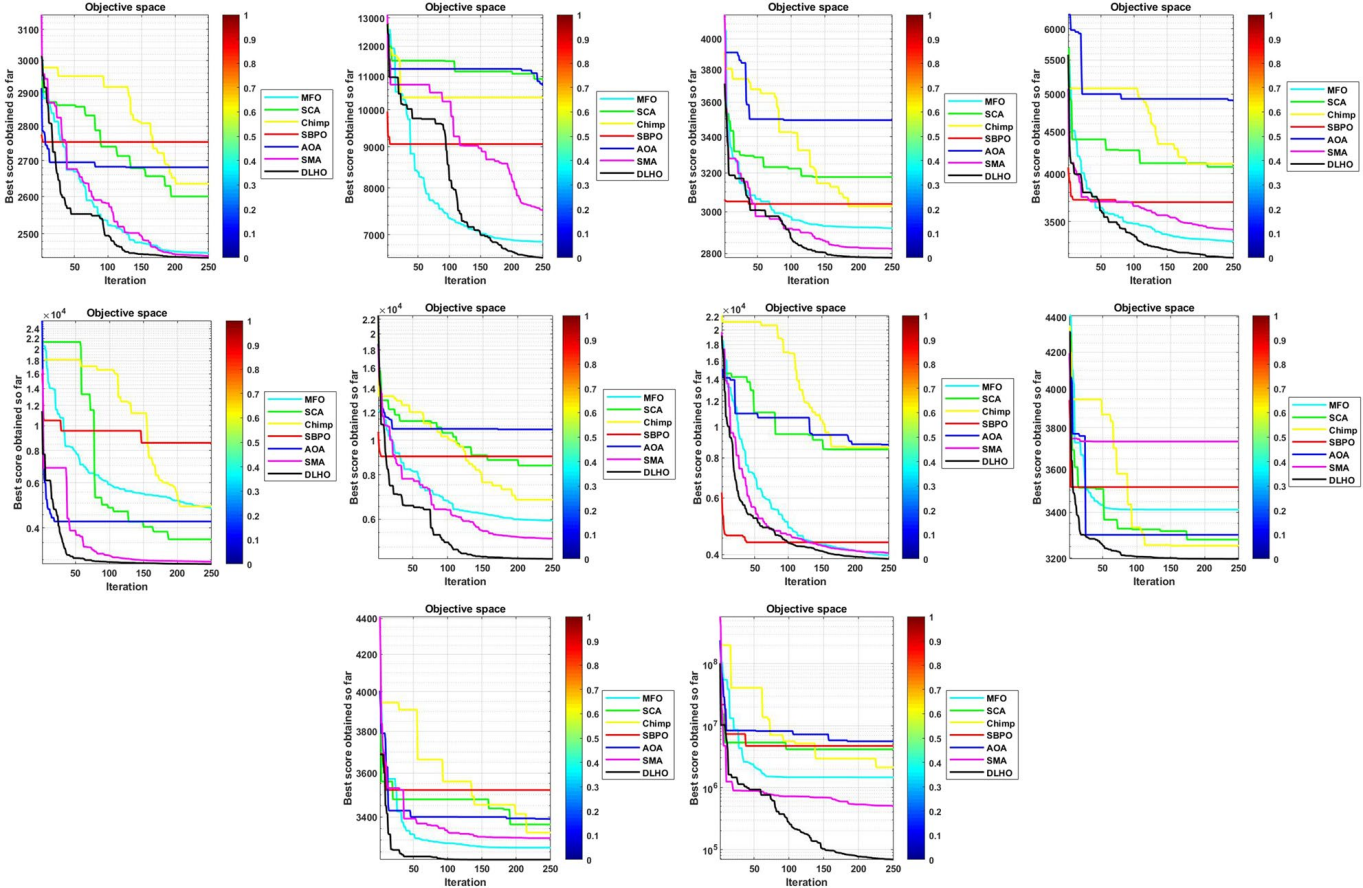
Performance graphs of Optimizers on composition test suites.

The performance graphs of the methods have been plotted between maximum number of generations and best outcomes so far, these shown in Figs. 4–7. These figures show that the best outcomes are obtained beside each generation which are shown by the best outcomes.

According to Berg et al.^40^, assures that the optimizers ultimately trace to a goal and catch the best outcomes in search areas. So, the DLHO expands the fitness outcome for every member and guarantees best goals for issues as generation increases. Now, it can be said that it happens by the enhancement of HHO method.Each member travels from highest to lowest optima and with this strategy the overall members are amends their locations with every generation.

With this strategy, the best outcomes are stored for searching the next best location for each member of the crowd in the search space. All these graphs show that the DLHO is competent in trapping the best outcomes for each function against the least number of generations which illustrates the fast convergence speed of the DLHO algorithm.

#### Assessment of robustness

The accuracy and robustness of the presented approach has been confirmed by utilizing the Wilcoxon signed ranks method for a superior assessment^41^.It is a non-parametric strategy that is applied on different samples for judging the significance difference amid them.The significance level shows the best sample amid them. However, this strategy generally helps in locating the significant difference of the behaviors of two algorithms.

The results of the Table 8 have been evaluated by the results of the Table 7. In the Table 8,if $p<1$, then it shows a rejection of the null hypothesis ($H_{0}$), whereas $p>1$, shows a failure to reject the ($H_{0}$).So, $p<0.05$, it shows that the proposed strategy is significantly better than the other algorithms.Other hand, if $p>0.05$, it presents that the attained enhancements are not statistically significant.

Here, in this phase for assessment the robustness of the present strategy the wilcoxon method has been utilized against the average values of the algorithms so that it could be concluded that the significant difference is amid the algorithms or not.In Table 8,it can be easily seen that the proposed strategy has better characteristics such as strength of the global optima goal and superiority of the optimal solution. Also, significant importance may be placed in local exploitation and global exploration.The Wilcoxon method results illustrated that the proposed strategy is superior among-others-in-comparison.Hence, the proposed strategy is-statistically superior and this has not happened by likelihood/or chance.

**Table 8:** Robustness assessment of the algorithms by Wilcoxon test.

Proposed method	Compared methods	$R^-$	$R^+$	Z value	P value	Accept ($H_{1}$)	Reject $H_{0}$
		-	-			($p<1$)	($p<1$)
–	MFO	71	0	–3.167	0.00154	Yes	Yes
–	SCA	77	0	–3.038	0.002382	Yes	Yes
–	Chimp	81	0	–2.951	0.003167	Yes	Yes
DLHO	SBPO	78	0	–3.016	0.002561	Yes	Yes
–	AOA	64	0	–3.319	0.000903	Yes	Yes
–	SMA	68	0	–3.232	0.001229	Yes	Yes

## Proposed technique(DLHO) for breast cancer detection

In this work, firstly the mammography pictures are fed into an MLP neural network (NN) for pre-processing to categorize the nonlinear distinguishable structures of the pictures. Secondly, preprocessed output has been passed to DLHO.The structure of the proposed methodology has been illustrated in following subsections:

### Picture pre-processing

Occasionally, Mammograms have a slight sound due to fluctuation and accidental alterations in dignified motions. Sound is a serious issue for picture-processing developments, mostly when boundaries inside the pictures have to be recognized, which needs separation. Separation rises the consequence of extraordinary frequency pixels, which comprises sound. In addition the median filter is utilized for the pre-processing procedure earlier picture division. it recalls boundaries although eliminating sound. It is a lowpass filter that needs a lengthier dispensation time than other filters. The median filter exchanges the particular pixel by the median of the nearby pixels. It is evaluate by mathematical is as follow;

24 $$\begin{aligned} y_{m,n}=M\left\{ x[i,j],\,\, \left( i,j \right) \epsilon \, \delta \right\} \end{aligned}$$

Where *M* illustrate the median and $\delta$ shows the neighbourhood mid nearby the position (*m*, *n*) in an picture.

### MLP neural networks (NNs)

**Figure 8:**
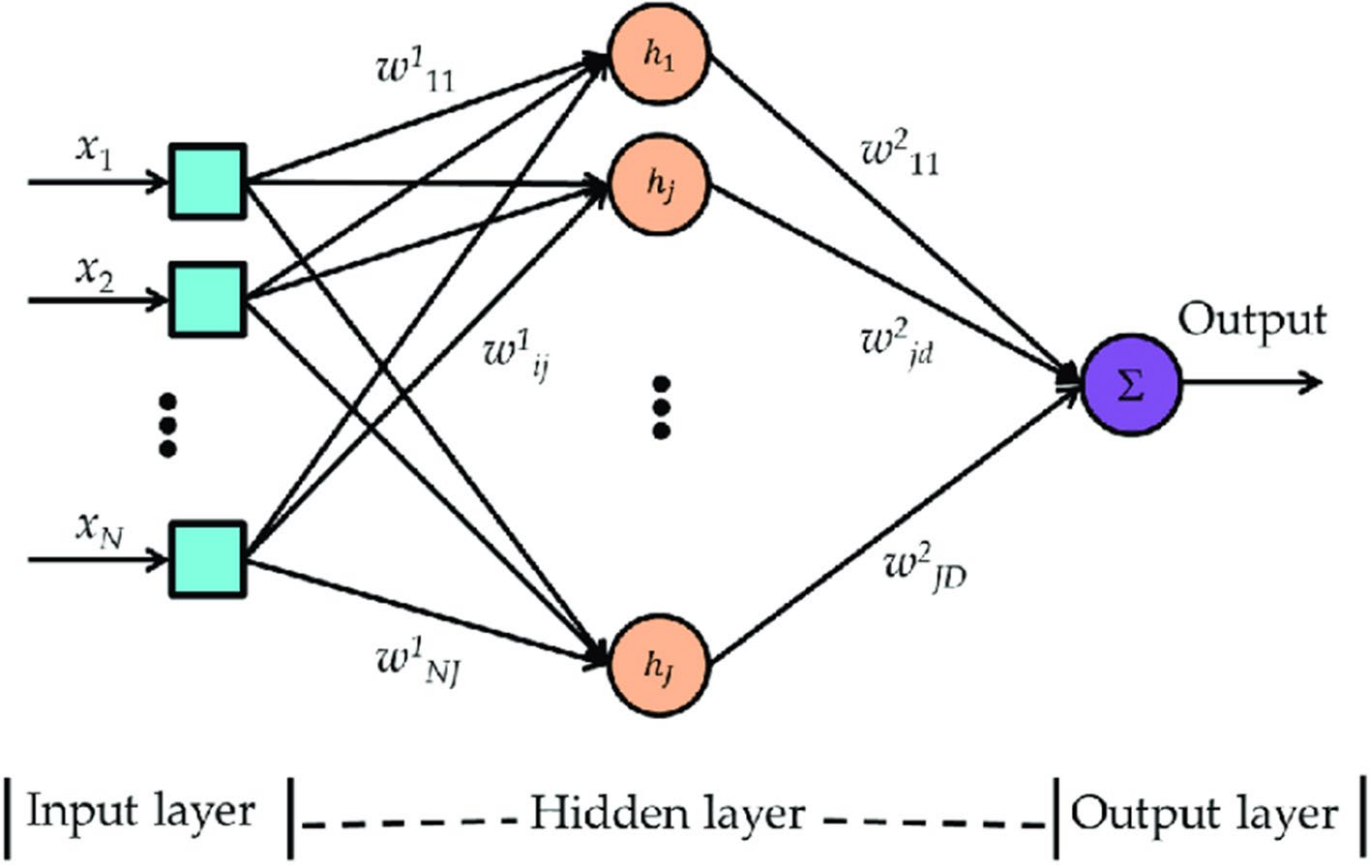
Basic structure of the MLP Neural Networks (NNs).

The NNs are unique of the extreme developments in the domain of artificial and computational intelligence. They mimic the neurons of human brain to frequently resolve the big databases related to bio-medical science. Every link amid the neurons is allocated a unusual weight, which specifies the amount of influence of the production on the contribution to the subsequent neuron. Usually, a neuron also has its individual weight, mentioned to as a bias, which defines the influence of the neuron on itself^42^.The basic structure of the MLP Neural Networks (NNs) has been illustrated by Fig. 8.

The outcome of every node can be calculated by two phases (1) firstly, the sum of the weight is calculated by the following equation;

25 $$\begin{aligned} z_{i}=\sum _{j=1}^{n}w_{ij}x_{i}+h_{j} \end{aligned}$$

Where $x_{i}$, $w_{ij}$ and $h_{j}$ represents the input, weight and hidden neuron *j*.Secondly, the activation function (AF) is utilized to evaluate the outcome of the neurons. During this study, the sigmoid function has been utilized in AF and it can be evaluate by the following equation;

26 $$\begin{aligned} f_{j}(x)=\frac{1}{1+e^{-z_{j}}} \end{aligned}$$

At end, the outcome of all neuron can be calculated by the following formula;

27 $$\begin{aligned} y_{i}=\sum _{j=1}^{m}w_{kj}x_{i}+h_{k} \end{aligned}$$

### DLHO based MLP trainer

The multilayer perceptron (MLP) plays an important role in the field of bio-medical science. Firstly, the optimizer method was utilized on these databases by. The weight and biases are the most important decision variables in these databases. Normally these variables are utilized for obtaining the superior prediction accuracy, approximation and classification respectively.Here the DLHO method accepts these decision variables in the form of a vector and it could be evaluate is as follows;

28 $$\begin{aligned} \mathbf {v}=\left\{ \overrightarrow{W},\overrightarrow{\theta } \right\} =\left\{ W_{1,1},W_{1,2},\dots ,W_{n,n},h,\theta _1,\theta _2,\dots ,\theta _h \right\} \end{aligned}$$

where *n*, $\theta _j$ and $W_{i,j}$ illustrates the number of input nodes, connection weight and bias of the $j^{th}$ node.

Further, the mean square error has been obtained by utilizing the following equation;

29 $$\begin{aligned} M_{error}=\sum _{i=1}^{m}\left( o_{i}^{k},d_{i}^{k} \right) ^2 \end{aligned}$$

where *m*, $o_{i}^{k}$ and $d_{i}^{k}$ illustrates the number of outcomes, desired outcome and actual outcome of the $i^th$ input. Now the accuracy of an multilayer perceptron (MLP) has calculated through mean square error over all the training trials by the following equation;

30 $$\begin{aligned} \overline{M_{error}}=\sum _{k=1}^{s}\frac{\sum _{i=1}^{m}\left( o_{i}^{k},d_{i}^{k} \right) ^2}{s} \end{aligned}$$

where *s* and *m* illustrates the number of training trails and number of outcomes respectively.At end, the fitness function has been obtained by the above equations is as follows;

31 $$\begin{aligned} Min\left( f(\mathbf {v}) \right) =\overline{M_{error}} \end{aligned}$$

### DLHO Algorithm and MSE

In this stage of implementation the proposed DLHO method has been applied to detect the breast cancer.Breast cancer detection is one of the major challenging well-known issue in the biomedical field. From last few decades, scientists of different fields are trying to solve this issue by various robust optimizers. Due to the complexities of this issue, a robust technique is needed so that we could tackle this problem. By merging the merits of two powerful techniques such as HHO and DHL^43^ search strategy, we have tried to present a new method for tackling this issue so that the best solution can be generated.

Metric MSE(mean square error) has been utilized to evaluate the difference between desired and actual outcomes for each sample or object. MSE can be calculated by the following equation;

32 $$\begin{aligned} MSE=\frac{1}{n}\sum _{i=1}^{n}\sum _{j=1}^{m}\left( y_{j}(k)-y_{j}^{*}(k) \right) ^{2} \end{aligned}$$

where $y_{j}(k)$ and $y_{j}^{*}(k)$ are denotes the actual and desire outcomes.The main objective of this study, the new hybrid version is utilization as a tool for breast cancer identification.

### Post-processing

During this work, the kapur’s strategy has been utilized for thresholding. Let *L* show the levels of a picture with *m* pixels. Then the average occurrence of a orange area *i* has been evaluated is as follows;

33 $$\begin{aligned} AO_{i}=\frac{k(i)}{m} \end{aligned}$$

where *k*(*i*) is illustrate the number of orange areas *i* in the picture.During the segmentation of the picture into various classes (*m*) we need $m-1$ outcomes. So limit of the orange levels for all class concerning optimal thresholds is defined as;

34 $$\begin{aligned} P_{i}:\left[ z_{i-1}^{*},\dots ,z_{i}^{*}-1 \right] \,\, \forall \,\, i=1,2,\dots ,m \end{aligned}$$

where $z_{0}=0$ and $z_{m}-1=L-1$.On the basis of above equation orange levels $w_{k}$ has been calculated by the following equation;

35 $$\begin{aligned} w_{k}=\sum _{j=z_{i-1}}^{z_{i}-1}P_{j} \end{aligned}$$

Where $w_{k}$ denote orange levels of $P_{i}$.Hence, image is segmented into two regions: R(0,z) and R(z,L)

36 $$\begin{aligned} Max\left( f(z) \right) =R(0,z)+R(z,L) \end{aligned}$$

where;

37 $$\begin{aligned} R(0,z)= & {} - \sum _{i=0}^{z-1}\left( \frac{P_{i}}{w_{0}}\times In \frac{P_{i}}{w_{0}} \right) ; \,\, w_{0}=\sum _{i=0}^{z-1}P_{i} \end{aligned}$$

38 $$\begin{aligned} R(z,L)= & {} - \sum _{i=z}^{L-1}\left( \frac{P_{i}}{w_{1}}\times In \frac{P_{i}}{w_{1}} \right) ; \,\, w_{1}=\sum _{i=z}^{L-1}P_{i} \end{aligned}$$

On the basis of above fitness function the optimum threshold has been obtained.

### Database report

**Figure 9:**
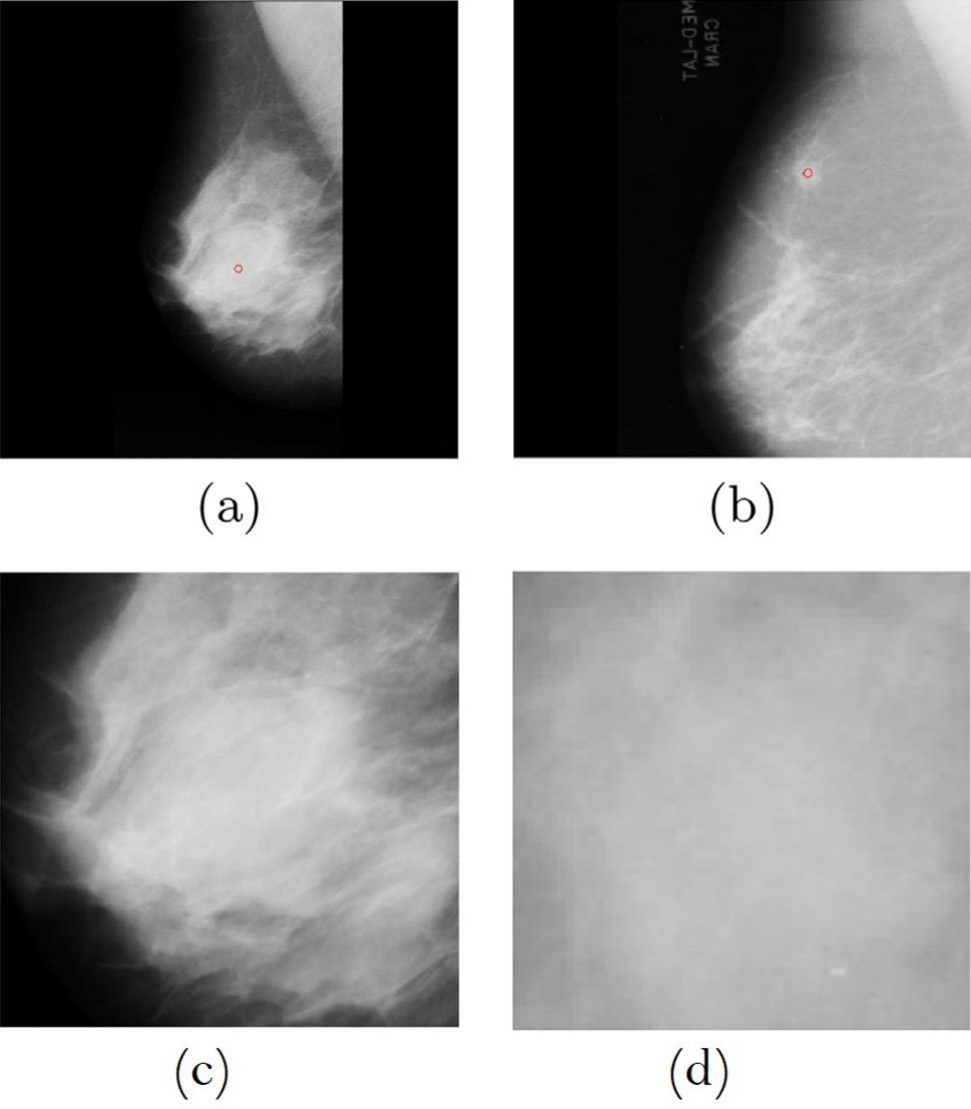
Sample mammogram image from the MIAS database.

To verify the applicability of the proposed method for breast cancer detection, experiments have been performed on following data sets:

- **First database** First MIAS database has been taken from the Pilot European Image Processing Archive (PEIPA) at the University of Essex^44^.It includes 322 digitized mammography pictures or images (Amid it contain 202 normal and 120 abnormal pictures) and size of each image is 1024 $\times$ 1024. The sample of these images are illustrated by Fig. 9.
- **Second database** Second database has been taken from the University of California at Irvine (UCI) Machine Learning Repository^45^.The weight and biases range has been fixed -10 to 10 for each database. The population size ($n=200$) and max number of generations ($M_{g}=250$) have been taken during the work.The details of input values are illustrated in Table 13.

### Results and discussion

Statistical and numerical computations are done on the first database that included 322 digitized mammographic images out of which 202 are normal and 120 are abnormal pictures and the size of this database is taken as 1024 $\times$ 1024. This work has been implement on Matlab R2018a with a 64-bit operating system, Core i3, 8th GEN and 8GB Ram respectively.

For verifying the robustness of the proposed method the obtained outcomes has been compared with recent algorithms such as BP^46^, GA^47^, PSO^48^, GWO^14^ and WOA^49^ respectively in terms of correct detection rate (CDR), false rejection rate (FRR) and false acceptance rate (FAR), mean and standard deviation etc. The CDR, FRR and FAR are calculated is as follows;

39 $$\begin{aligned} D_{r}=\frac{C_{p}}{T_{p}} \end{aligned}$$

where $D_{r}$,$C_{p}$ and $T_{p}$ are illustrates the correct detection rate, number of pixels correctly classified and total number of pixels in the database respectively.

40 $$\begin{aligned} F_{a}=\frac{H_{p}}{T_{p}} \end{aligned}$$

where $$ F_{a}$$,$$H_{p}$$ and $$T_{p}$$ denote the false acceptance rate, number of healthy pixels classified as breast cancer and total number of pixels in the database.

41 $$\begin{aligned} F_{r}=\frac{C_{p}}{T_{p}} \end{aligned}$$

where $$ F_{r}$$,$$C_{p}$$ and $$T_{p}$$ denote the false rejection rate, number of cancer pixels classified as healthy and total number of pixels in the database.

**Figure 10:**
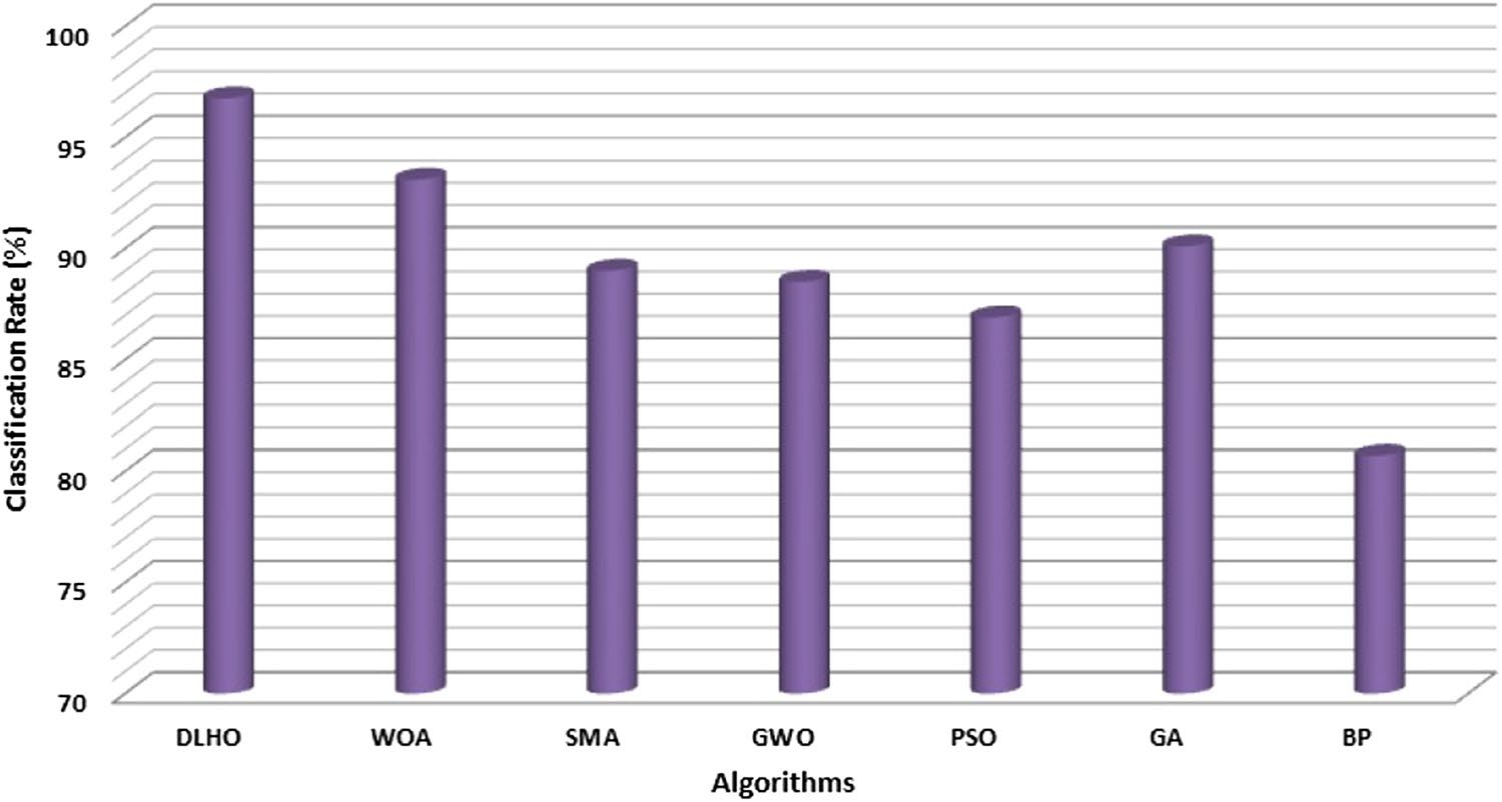
Cancer detection rate by algorithms on MIAS database.

Experimental outcomes of the algorithms on the MIAS database have been illustrated through Table 9 and Fig. 10.The graph is plotted (Fig. (10)) to compare the cancer detection rate (%) of various algorithms.

**Table 9:** Comparison of different algorithms on MIAS database in terms of different metrics.

Metric (%)	DLHO	WOA	SMA	GWO	PSO	GA	BP
CDR	96.76	93.1	89.36	88.5	86.9	90.1	80.7
FAR	2.98	3.1	5.9	6.5	5.9	11.5	7.9
FRR	2.08	2.8	5.5	6.5	4	7.7	6.3
$\sigma$	3.89E–03	7.15E–03	1.03E–02	2.85E–02	3.99E–02	1.93E–01	4.87E–02
$\mu$	93.54	93.1	88.5	86.9	90.1	80.7	91.8

**Figure 11:**
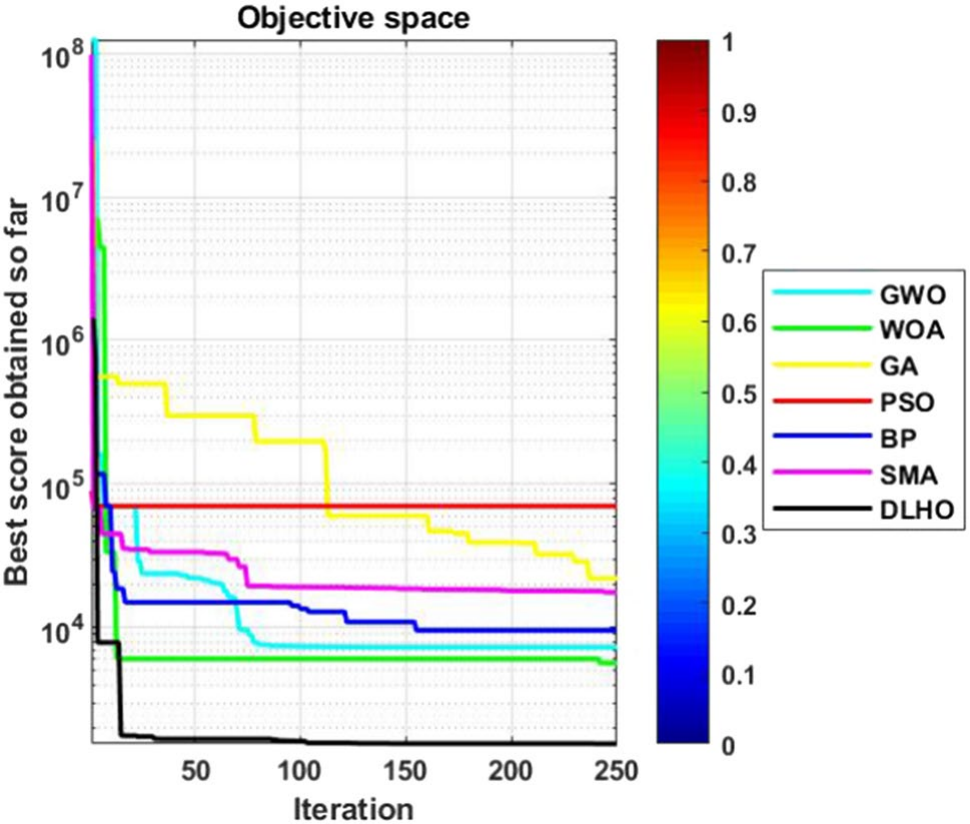
MSE graphs of algorithms on first database for cancer detection.

**Table 10:** Computational time of algorithms on MIAS database for cancer detection.

Metric (%)	DLHO	WOA	SMA	GWO	PSO	GA	BP
C-time	9.78	22.46	23.67	27.19	20.02	26.86	10.16

**Figure 12:**
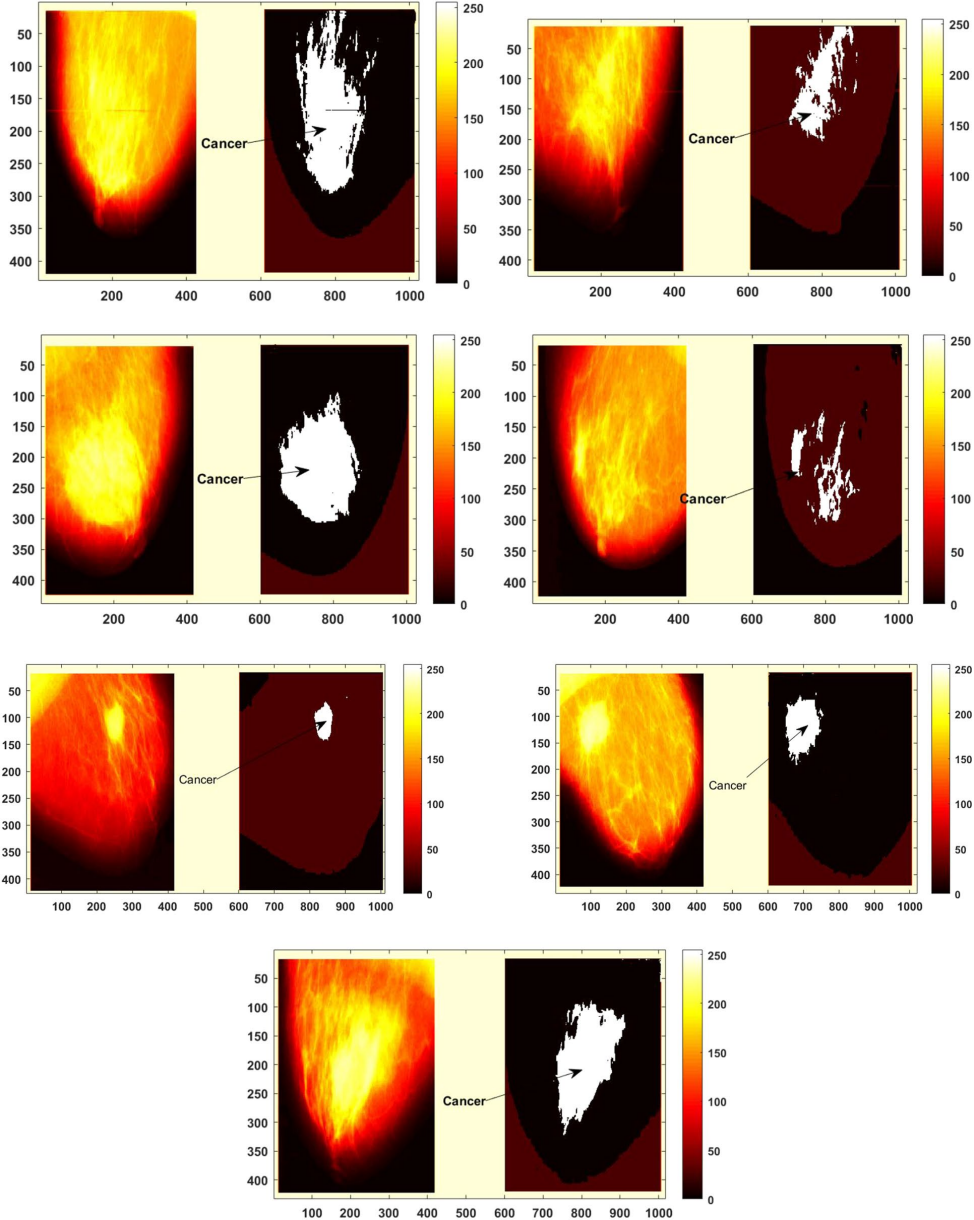
Cancer detection by DLHO algorithm.

**Figure 13:**
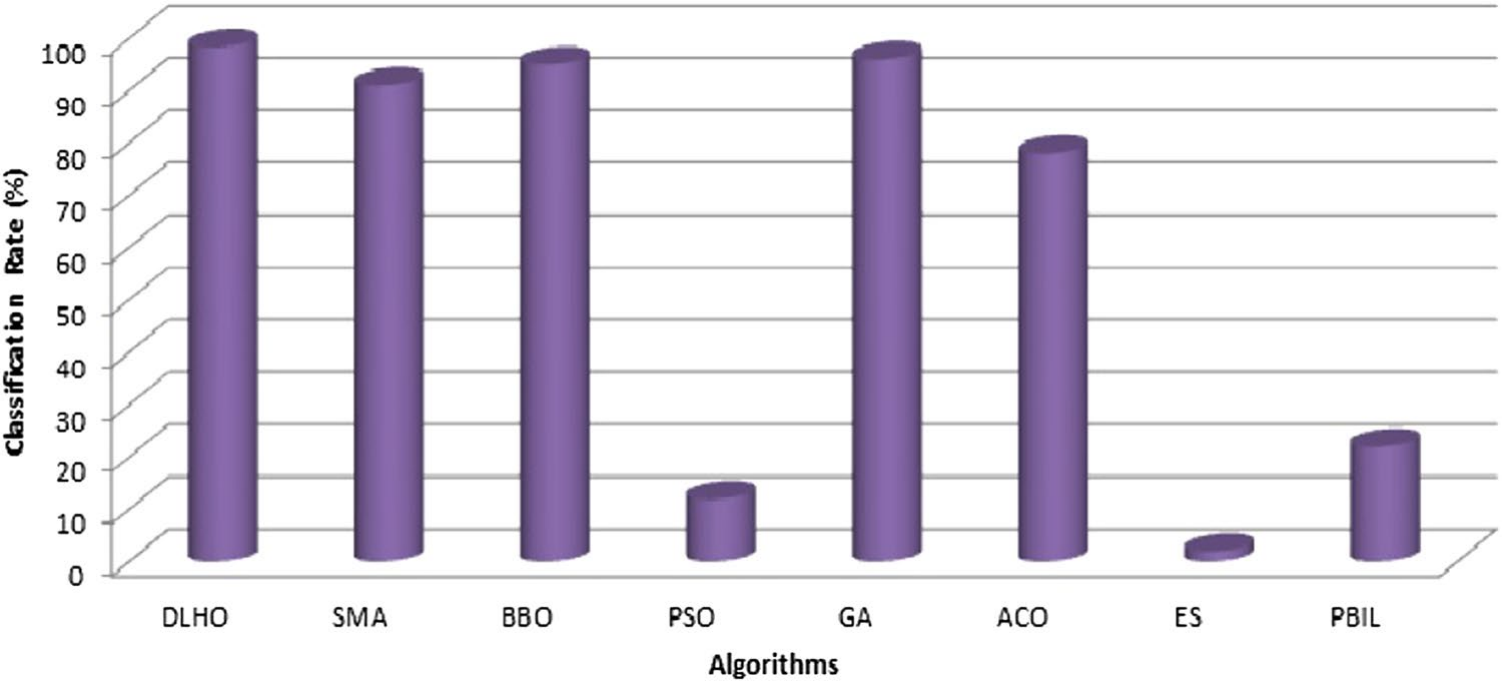
Breast Cancer detection Rate (%) of algorithms on second database.

During the implementation of the proposed algorithm on the second database for breast cancer,various input parameters are set such as number of attributes (9), number of training objects (599), number of test objects (100) and number of classes (2) respectively. The algorithm is run iteratively to find the best optima.Tabulated outcomes of the methods are illustrated in the Table 11. The classification rate of the methods against this database are drawn by Fig. 13. Figure (12) depicts the detection of left and right breast cancer detection in eight mammogram pictures using the DLHO algorithm.

Again the values in the Tables 9–11, show the superiority of the proposed method.The statistical score shows that DLHO method has a high ability to ignore local optima and approximate the superior global optima outcomes for biases and weights.This issue has the utmost complexity compared to the earlier discussed database issues in terms of the biases, weights and training objects. Experimental solutions give strong evidence of the suitability of the DLHO method in training multi-layer perceptions.The proposed method illustrates the high local prevention.For alternative, the local search around to global optimum and exploitation are high.

**Table 11:** Algorithms outcomes for the Breast Cancer detection on second database.

Method	$\mu$	$\sigma$	CR(%)
DLHO	0.0012	3.1012e–06	98.39
SMA	0.0078	8.7612e–02	91.00
BBO	0.0079	0.0089	95.00
PSO	0.0457	0.0357	12.00
GA	0.0082	0.0100	96.00
ACO	0.0228	0.0106	78.00
ES	0.0381	0.0023	2.00
PBIL	0.0366	0.0053	22.00

**Table 12:** Computational time of algorithms on second database for cancer detection.

Metric (%)	DLHO	SMA	BBO	PSO	GA	ACO	ES	PBIL
C-time	10.02	17.99	16.09	16.33	13.87	21.34	18.76	29.88

Here noticeably the cancer detection rate (%) of the maximum optimizer on this issue is very poor due the complexity of this data set.However, all the methods have been tested on the same parameter settings so that we can correctly find out the robustness of the algorithms.Tabulated values of the methods strongly shows that the proposed method is competent to solve this problem with best optima, accuracy and high rate of cancer detection as compared to others.The computational time of the algorithms for cancer detection have been reported in the Tables 10–12. All the values in the tables have proved that the proposed method is competent to detect the cancer with highest detection rate as well as have least computational time as compared to others.

**Figure 14:**
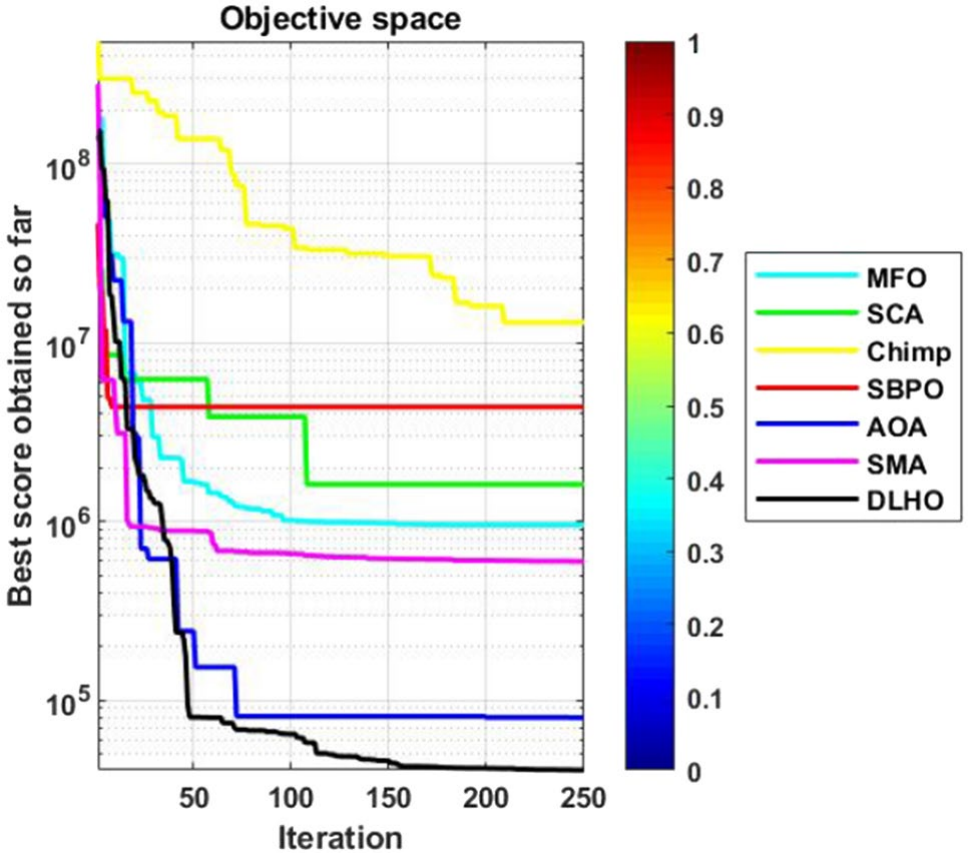
MSE graphs of algorithms on second database for cancer detection.

Figures 11–14, are shows the mean square error of the algorithm on the first and second database. As stated, the metaheuristics were run various times to confirm the robustness. These graphs show that the proposed method successfully solves these databases with least mean square errors.

By experiments, it could be determined that a huge drawback of the recent algorithms is that numerous weaknesses such as getting stuck at a local optima, slow convergence, weak exploration and exploitation balance and premature convergence cause them to fail to resolve complex issues in certain cases (see Figs. 11–14).

## Robustness of DLHO for other biomedical databases

In this part of research, two different bio-medical issues such as balloon and heart are taken from the University of California at Irvine (UCI) Machine Learning Repository^45^. On these databases various population based optimizers are applied^14,50–52^ for evaluating the high rate of classification and accuracy of the outcomes.The weight and biases range has been fixed -10 to 10 for each database. The crowd size is set 50 for Balloon, 200 for the rest databases and 250 maximum number of generations have taken during the implementation of the codes.

The input values for these databases have been illustrated by Table13. Each method has been run various times to generate the best outcomes for each databases. The outcomes of the optimizers have been presented in the terms of average, standard and classification rate (%) respectively. Noticeably, the least mean and standard score of mean square error in the end of the generation shows superior performance and accuracy.

The normalization used in this work is called min-max Normalization which is the most important stage of MLP when we solve these databases with attributes in different sizes or ranges. During this work, the stage is known as min and max normalization and is evaluate is as follows;

42 $$\begin{aligned} X^*=\frac{(x-l)(n-o)}{(m-l)}+o \end{aligned}$$

where the above mathematical equation maps *x* in the range of [*l*, *m*]-[*n*, *o*].

**Table 13:** Classification databases^14^.

Problem	No’s of	No’s of	No’s of test	No’s of
-	attributes	training objects	objects	classes
Ballon	4	16	16	2
Breast Cancer	9	599	100	2
Heart	22	80	187	2

### Balloon

Balloon database has been tested through the algorithms on different settings of samples such as number of attributes (4), number of training objects (16), number of test objects (16) and number of classes (2) respectively. Experimental outcomes of the algorithms have been reported through table 14.

On the basis of experimental outcomes, firstly we can seen that the classification rate of each algorithm is same, however the average and standard scores of the algorithms for this database are unique. The least average and standard score shows the accuracy, robustness and stability of the algorithms for the functions. So on the basis of these outcomes we can concluded that the DLHO algorithm is competent to provide the high accurate, robustness and stability performance of this issues than others. The statistical outcomes proved the robustness of the proposed method.

**Table 14:** Algorithms outcomes for the balloon database.

Method	$\mu$	$\sigma$	CR(%)
DLHO	1.89e–34	4.89e–30	100
SMA	5.89e–011	6.34e–9	100
MGWO^16^	0.0014	0.0132	100
GWO^14^	9.38e–15	2.81e–14	100
PSO^14^	0.000585	0.000749	100
GA^14^	5.08e–24	1.06e–23	100
ACO^14^	0.004854	0.007760	100
Es^14^	0.019055	0.170260	100
PBIL^14^	2.49e–05	5.27e–05	100

### Heart

**Figure 15:**
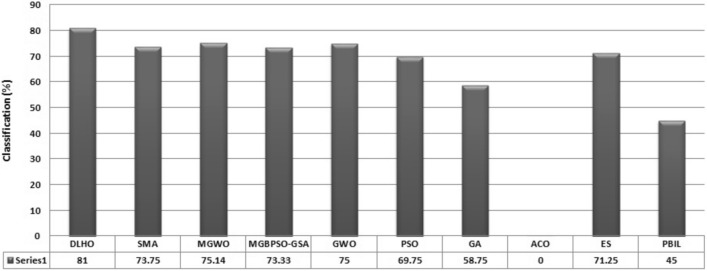
Classification Rate (%) of algorithms on Heart database.

In lastly, the heart database has been solved by the optimizer methods which have number of attributes (22), number of training objects (80), number of test objects (187) and number of classes (2). Multi-layer perceptron’s against the construction of 22-45-1 has been trained through the metaheuristics. Obtained outcomes of the metaheuristics have been reported by table 15 and the classification rate of the algorithms on the heart database are plotted by Fig. 15.

Experimental outcomes revealed that the DLHO is competent at giving the superior classification rate with the best statistical outcomes. Here, it could be concluded that the proposed method most effective in issue approximation heart database. The least mean and standard deviation outcomes reveals the better local optima avoidance of the proposed method.All simulation also illustrates that the superior error fits the proposed method. This validates the accuracy and best performance of the DLHO method as well.

**Table 15:** Algorithms outcomes for the Heart database.

Method	$\mu$	$\sigma$	CR(%)
DLHO	0.01512	0.000189	81.00
SMA	0.16789	0.009435	73.75
MGWO^16^	0.0765	0.0376	75.14
MGBPSO-GSA^15^	0.10442	0.002041	73.33
GWO^14^	0.122600	0.007700	75
PSO^14^	0.188568	0.008939	69.75
GA^14^	0.093047	0.022460	58.75
ACO^14^	0.228430	0.004979	00
ES^14^	0.192473	0.015174	71.25
PBIL^14^	0.154096	0.018204	45

## Conclusion

This work points out an enhanced version with the integration of merits of Harris Hawks Optimization (HHO) algorithm and dimension learning based hunting (DLH) search strategy for biomedical issues, it is called DLHO algorithm. In this modification the DLH search strategy has been utilized for enhancing the exploration and exploitation stages of the HHO algorithm, so that the above weakness could be removed. For verification of effectiveness the DLHO method has utilized 29 standard suites and three database. However, the robustness of the classifiers has been verified on all features and particular attributes distinctly to get and compare the attained accuracy. All simulations reveal that the presented algorithm is able i) to detect the high quality of global solutions and ii) to define the effective optima outcomes for biases and weight in terms of local avoidance and detection or classification rate or produces high accuracy for the biomedical issues as compared to others.

For future work, we shall present a more enhanced version for biomedical and engineering application. At end, we believe this research will inspire every young scientist, who is recently working on meta-heuristics and engineering applications.
